# Impact of ventilation strategies on pulmonary and cardiovascular complications in patients undergoing general anaesthesia for elective surgery: a systematic review and meta-analysis

**DOI:** 10.1016/j.bja.2023.09.011

**Published:** 2023-10-14

**Authors:** Pasquale Buonanno, Annachiara Marra, Carmine Iacovazzo, Maria Vargas, Antonio Coviello, Francesco Squillacioti, Serena Nappi, Andrea Uriel de Siena, Giuseppe Servillo

**Affiliations:** Department of Neuroscience, Reproductive Science and Odontostomatological Science, University of Naples ‘Federico II’, Naples, Italy

**Keywords:** driving pressure, general anaesthesia, positive end-expiratory pressure, postoperative pulmonary complications, ventilation strategy

## Abstract

**Background:**

Many RCTs have evaluated the influence of intraoperative tidal volume (tV), PEEP, and driving pressure on the occurrence of postoperative pulmonary complications, cardiovascular complications, and mortality in adult patients. Our meta-analysis aimed to investigate the association between tV, PEEP, and driving pressure and the above-mentioned outcomes.

**Methods:**

We conducted a systematic review and meta-analysis of RCTs from inception to May 19, 2022. The primary outcome was the incidence of postoperative pulmonary complications; the secondary outcomes were intraoperative cardiovascular complications and 30-day mortality. Primary and secondary outcomes were evaluated stratifying patients in the following groups: (1) low tV (LV, tV 6–8 ml kg^−1^ and PEEP ≥5 cm H_2_O) *vs* high tV (HV, tV >8 ml kg^−1^ and PEEP=0 cm H_2_O); (2) higher PEEP (HP, ≥6 cm H_2_O) *vs* lower PEEP (LP, <6 cm H_2_O); and (3) driving pressure-guided PEEP (DP) *vs* fixed PEEP (FP).

**Results:**

We included 16 RCTs with a total sample size of 4993. The incidence of postoperative pulmonary complications was lower in patients treated with LV than with HV (OR=0.402, CI 0.280–0.577, *P*<0.001) and lower in DP than in FP group (OR=0.358, CI 0.187–0.684, *P*=0.002). Postoperative pulmonary complications did not differ between HP and LP groups; the incidence of intraoperative cardiovascular complications was higher in HP group (OR=1.385, CI 1.027–1.867, *P*=0.002). The 30-day mortality was not influenced by the ventilation strategy.

**Conclusions:**

Optimal intraoperative mechanical ventilation is unclear; however, our meta-analysis showed that low tidal volume and driving pressure-guided PEEP strategies were associated with a reduction in postoperative pulmonary complications.

**Editor's key points**

• Intraoperative mechanical ventilation can influence the incidence of postoperative pulmonary and cardiovascular complications; however, the optimal ventilatory strategy remains unclear.
• In this meta-analysis, the authors found that low tidal volume and driving pressure-guided PEEP were associated with a reduction in postoperative pulmonary complications.
• This evidence suggests that patients receiving mechanical ventilation should be provided with 6–8 ml kg^−1^ tidal volume and low PEEP (to reach the lowest driving pressure). Future research is needed to confirm the relationship of ventilator settings with postoperative outcomes.

Every year, more than 230 million people need invasive mechanical ventilation during general anaesthesia for surgical procedures.^1^ Many studies have evaluated the influence of tidal volume (tV), PEEP, and driving pressure on the occurrence of postoperative pulmonary complications (PPCs), cardiovascular complications (CVCs), and mortality, but the best intraoperative ventilatory setting is far from being established.^2^, ^3^, ^4^, ^5^, ^6^, ^7^

Two modalities of mechanical ventilation are usually identified: the conventional ventilation also called high tV (HV) ventilation (>8 ml kg^−1^ of predicted body weight) with a low or zero PEEP (ZEEP) and the lung protective ventilation or low tV (LV) ventilation (6–8 ml kg^−1^ of predicted body weight) usually associated with a higher PEEP level.^8^ These ventilation techniques were deeply investigated in the management of acute respiratory distress syndrome (ARDS) in the ICU and, later, they were applied to the mechanical ventilation in the operating room, but which of them could reduce perioperative complications remains a matter of debate.

The HV ventilation has shown to reduce hypoxaemia and atelectasis, but it is also associated with a higher incidence of lung injuries (acute lung injury), overdistention, and inflammation, whereas the LV ventilation has shown to reduce mortality in patients with ARDS, but it can cause alveolar collapse and atelectrauma if not associated to the best PEEP.^6^^,^^7^^,^^9^ The calculation of the best PEEP is still a challenge, and several strategies were studied for titrating the best level of PEEP.^10^

In recent years, the LV ventilation was suggested to reduce PPCs and to improve intraoperative oxygenation even in the setting of general anaesthesia for elective surgery^11^; however, higher PEEP level can cause alveolar overdistention and haemodynamic instability.^12^ The aim of our meta-analysis is to determine the effects of different types of intraoperative mechanical ventilation settings on PPCs, CVCs, and 30-day mortality.

## Methods

This systematic review and meta-analysis followed the statement guidelines of Preferred Reporting Items for Systematic Reviews and Meta-Analyses (PRISMA), and was registered in the PROSPERO database (CRD42022334241).^13^

We performed a comprehensive search in the following databases: Medline, Cumulative Index to Nursing and Allied Health Literature (CINAHL), Web of Science, and Cochrane Central Register of Controlled Trials (CENTRAL) from inception to May 19, 2022, using the search string “(protective ventilation OR lower tidal volume OR low tidal volume OR positive end-expiratory pressure OR positive end expiratory pressure OR PEEP) AND (surgery OR surgical OR intraoperative OR anesthesia OR anaesthesia)”.

Inclusion criteria were: age >18 yr, RCTs, English language, elective surgery. Exclusion criteria were: pregnant women, patients with history of severe chronic pulmonary disease (COPD, uncontrolled bronchial asthma, severe restrictive lung disease), pulmonary metastases, cardiac and thoracic surgery, need for chest drainage prior to surgery, preoperative renal replacement therapy, congestive heart failure (NYHA: Class III or IV), one-lung intraoperative ventilation, and studies with missing data.

Two authors (FS and SN) independently assessed eligibility based on the titles, abstracts, full-text reports, and further information were acquired from investigators if needed; disagreements were solved by discussion.

Data extraction was performed with a standardised electronic data sheet to summarise information. Two authors (AUDS and PB) independently assessed the risk of bias of each study using the Cochrane Risk of Bias (RoB2) tool^14^; we assigned a value of ‘low risk’, ‘some concerns’, and ‘high risk’ of bias for the following domains: randomisation process, deviation from the intended interventions, missing outcome data, measurement of the outcome, and selection of the reported result. Disagreements were resolved by consensus.

The primary outcome was the incidence of PPCs: hypoxaemia (defined as *P*aO_2_ <60 mm Hg or SpO_2_ <90%), bronchospasm, development of ARDS, pulmonary infection, and radiological findings (chest radiography, CT scan, or lung ultrasound) of pulmonary infiltrate, aspiration pneumonitis, atelectasis, pleural effusion, pulmonary oedema, and pneumothorax.

The secondary outcomes were intraoperative hypotension (systolic blood pressure less than 90 mm Hg), hypertension (mean arterial blood pressure >90 mm Hg), bradycardia (HR <50 beats min^−1^ or a decrease of more than 20% from baseline), tachycardia (HR >95 beats min^−1^, any new arrhythmia, need of vasoconstrictor and inotropic drugs, non-fatal myocardial infarction, and 30-day mortality.

Primary and secondary outcomes were evaluated stratifying patients in the following groups: (1) LV (defined as tV=6–8 ml kg^−1^ and PEEP ≥5 cm H_2_O) *vs* HV (defined as tV >8 ml kg^−1^ and ZEEP); (2) higher PEEP (HP, ≥6 cm H_2_O) *vs* lower PEEP (LP, <6 cm H_2_O); (3) driving pressure-guided PEEP (DP) *vs* fixed PEEP (FP). After an overall evaluation, a sensitivity analysis was performed, excluding studies with high risk of bias. Moreover, a subgroup analysis was conducted to investigate the incidence of PPCs in obese and non-obese patients. Dichotomous variables were reported as odds ratio (OR) with 95% confidence intervals (CIs). Differences were considered statistically significant when *P-*value was <0.05. Heterogeneity was assessed by I^2^ statistic. Heterogeneity is a measure of clinical and methodological diversity among the studies; a corresponding low *P*-value (<0.05) indicates the presence of significant heterogeneity of intervention effects. I^2^ represents the percentage of the variability of the estimated effect because of the heterogeneity itself and not to chance, and according to its value, heterogeneity can be defined as low if I^2^ is less than 25%, moderate if it ranges from 25% to 75%, and high if more than 75%.^15^ Statistical meta-analysis was performed using Open Meta [Analyst] software.^16^

We calculated the fragility index using the calculator tool ClinCalc: Fragility Index Calculator to assess the robustness of each study; furthermore, we reported the median and the range of the fragility indices of the enrolled studies.^17^

A trial sequential analysis (TSA) was performed for each outcome using TSA software-v. 0.9.5.10 to establish if the meta-analysis is conclusive or more studies are needed.^18^

## Results

**Fig 1:**
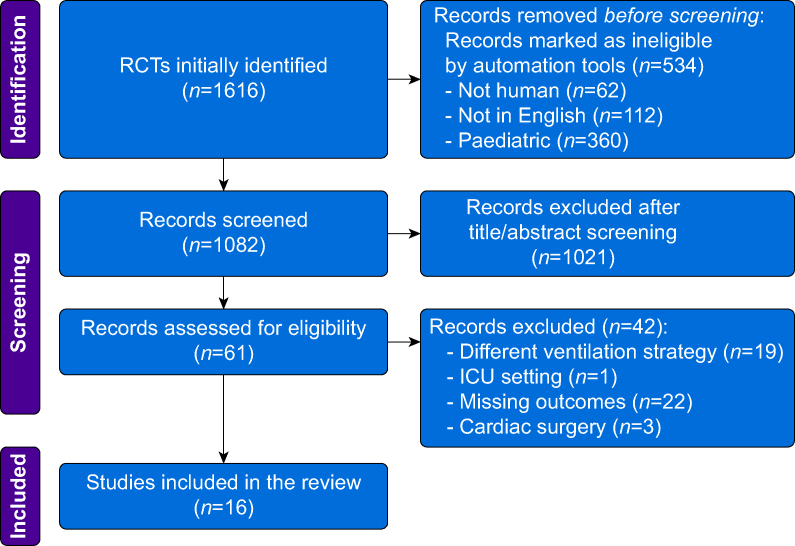
PRISMA flowchart.

We selected 16 studies with a total sample size of 4993 (Supplementary Table S1).^5^^,^^10^, ^11^, ^12^^,^^19^, ^20^, ^21^, ^22^, ^23^, ^24^, ^25^, ^26^, ^27^, ^28^, ^29^, ^30^ Figure 1 shows the PRISMA flow chart: 1616 RCTs were identified; 534 records were excluded by automation tools; 1021 articles were excluded by title and abstract; 42 studies were excluded by different outcomes, ventilatory strategies, settings, or missing data.

**Fig 2:**
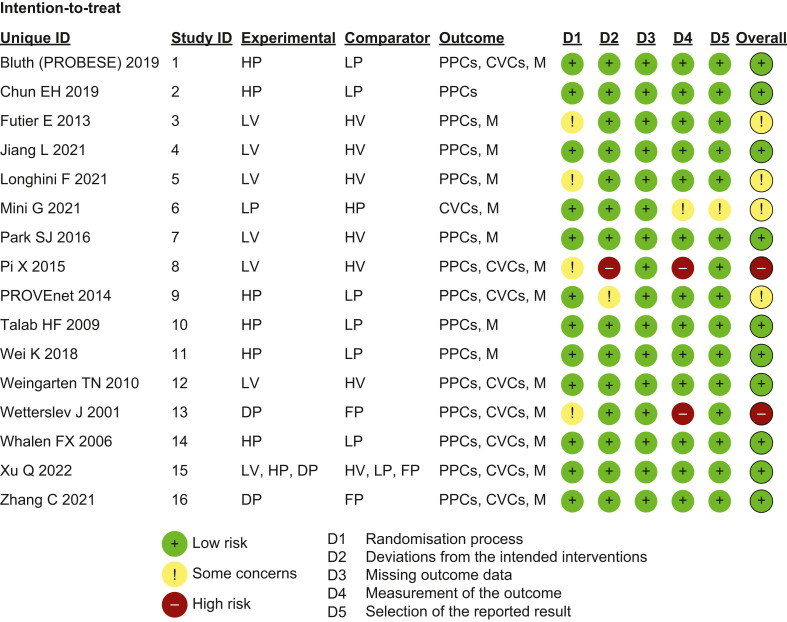
Risk of bias assessment. HP: higher positive end-expiratory pressure (PEEP); LP: lower PEEP; PPCs: postoperative pulmonary complications; CVCs: cardiovascular complications; M: mortality; LV: low tidal volume (tV) ventilation; HV: high tV ventilation; DP: driving pressure-guided PEEP; FP: fixed PEEP.

Figure 2 reports the risk of bias. Ten studies had a low overall risk of bias; four studies were globally evaluated with some concerns on the basis of an unclear randomisation process and outcome analysis; two studies were classified as having a high overall risk of bias mainly because of the randomisation process and the deviation from intended interventions.

### Postoperative pulmonary complications

**Fig 3:**
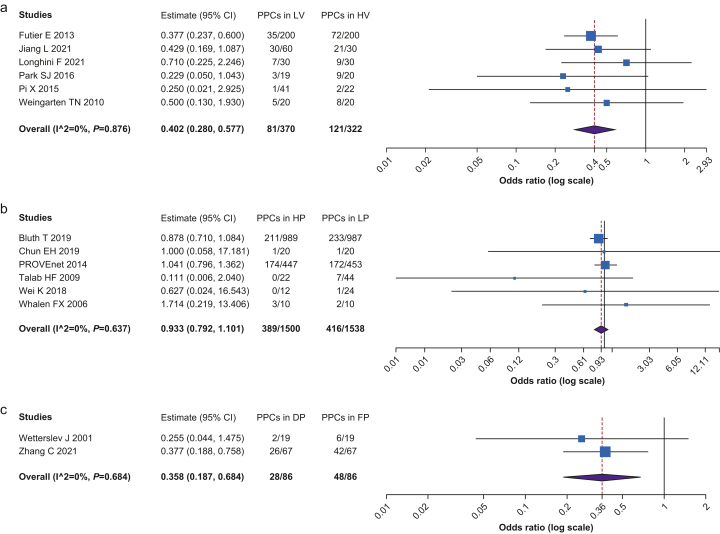
Postoperative pulmonary complications among the different ventilation strategies. (a) Comparison between low tidal volume (LV) and high tidal volume (HV) strategies. (b) Comparison between higher PEEP (HP) and lower PEEP (LP) strategies. (c) Comparison between driving pressure-guided PEEP (DP) and fixed PEEP (FP) strategies.

Figure 3 shows the analysis of PPCs. The patients treated with LV showed a lower probability of PPCs than those treated with HV strategy (*n*=692, OR=0.402, CI 0.280–0.577, *P*<0.001, I^2^=0, Het. *P*=0.876). Patients with a DP strategy showed a lower risk of PPCs in contrast with the FP strategy (*n*=172, OR=0.358, CI 0.187–0.684, *P*=0.002, I^2^=0, Het. *P*=0.684). There was no difference in the risk of PPCs between HP and LP strategies.

### Cardiovascular complications

**Fig 4:**
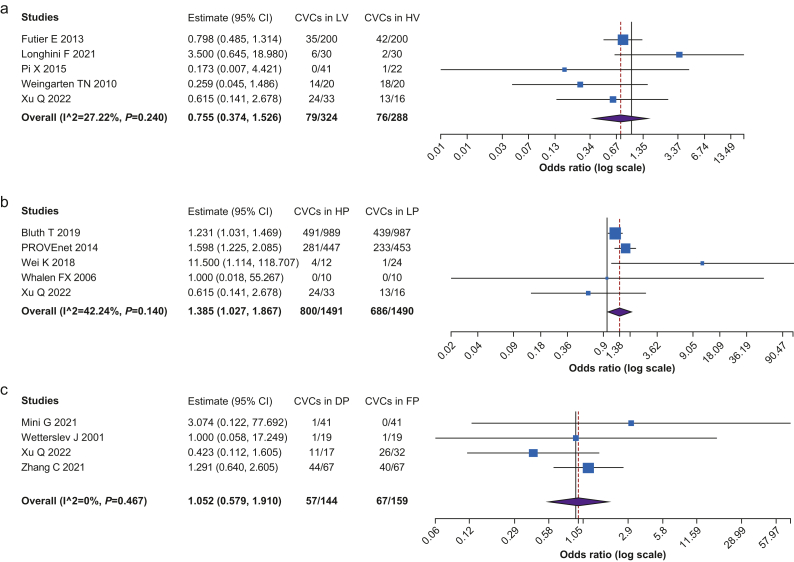
Cardiovascular complications among the different ventilation strategies. (a) Comparison between low tidal volume (LV) and high tidal volume (HV) strategies. (b) Comparison between higher PEEP (HP) and lower PEEP (LP) strategies. (c) Comparison between driving pressure-guided PEEP (DP) and fixed PEEP (FP) strategies.

Figure 4 shows the comparison of CVCs events among considered ventilation strategies. The HP group showed a higher risk of CVCs than the LP group (*n*=2981, OR=1.385, CI 1.027–1.867, *P*=0.002, I^2^=42.239, Het. *P*=0.140). There were no significant statistical differences in CVCs between LV and HV groups and between the DP and FP groups.

### Mortality

**Fig 5:**
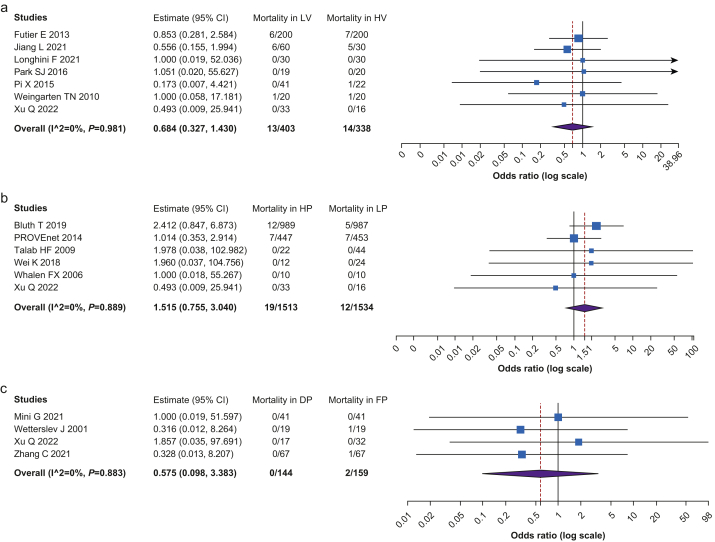
Mortality among the different ventilation strategies. (a) Comparison between low tidal volume (LV) and high tidal volume (HV) strategies. (b) Comparison between higher PEEP (HP) and lower PEEP (LP) strategies. (c) Comparison between driving pressure-guided PEEP (DP) and fixed PEEP (FP) strategies.

Figure 5 shows the comparison of rate of mortality among the groups. No significant statistical differences were recorded between the HV and LV groups (*n*=741, OR=0.684, CI 0.327–1.430, *P*=0.312, I^2^=0, Het. *P*=0.981), HP and LP groups (*n*=3047, OR=1.515, CI 0.755–3.040, *P*=0.242, I^2^=0, Het. *P*=0.889), and DP and FP groups (*n*=303, OR=0.575, CI 0.098–3.383, *P*=0.541, I^2^=0, Het. *P*=0.883).

### Sensitivity and subgroup analysis

Supplementary Figure S1 reports the results of sensitivity analysis. The studies with high risk of bias were involved in the comparison between HV and LV groups and between DF and FP groups. The sensitivity analysis confirmed that the risk of PPCs was higher in the HV group than in the LV group (*n*=629, OR=0.406, CI=0.282–0.585, *P*<0.001, I^2^=0, Het. *P*=0.799). The sensitivity analysis of PPCs between DP and FP groups was not performed as only one study presented a low risk of bias. No differences were recorded in the incidence of CVCs and mortality between HV and LV groups and between DP and FP groups.

Supplementary Figure S2 reports the subgroup analysis of the bariatric and non-bariatric studies. Obese patients were enrolled only in studies comparing HP and LP strategies. The risk of CVCs was higher in bariatric patients (*n*=2925, OR=1.345, CI=1.048–1.726, *P*=0.02, I^2^=44.78, Het. *P*=0.163) ventilated with HP strategy than with LP strategy. No differences were reported in PPCs and mortality in both bariatric and non-bariatric patients treated with HP or LP strategies.

### Fragility index and trial sequential analysis

The fragility indices of each study and their median and range were reported in Supplementary Table S2, which shows that the enrolled studies are far from being robust.^31^ The results of TSA for overall analysis are reported in Supplementary Figure S3: TSA shows that our meta-analysis is conclusive only for the evaluation of the risk of PPCs (Supplementary Fig. S3a–c). The meta-analysis suggests that HP is probably linked with a greater risk of CVCs than LP, but more studies are needed (Supplementary Fig. S3e).

Supplementary Figure S4 reports TSA for sensitivity analysis: it shows that the results of our meta-analysis about PPCs remain conclusive, even excluding studies with a high risk of bias (Supplementary Fig. S4a), whereas other studies are needed to clarify the impact of LV vs HV and DP vs FP on CVCs and mortality (Supplementary Fig. S4b–e).

Supplementary Figure S5 reports the TSA for the subgroup analysis of studies on bariatric and non-bariatric patients comparing HP and LP strategies. The subgroup meta-analysis for CVCs in non-obese patients reached the estimated sample size and, consequently, the results can be considered conclusive (Supplementary Fig. S5d), whereas the results about CVCs in obese patients and PPCs and mortality for both obese and non-obese patients are not definitive (Supplementary Fig. S5a–c, e, and f).

## Discussion

Our systematic review and meta-analysis showed that intraoperative low tidal volume and low driving pressure-guided PEEP reduce postoperative pulmonary complications compared with high tidal volume and fixed PEEP ventilation strategies; higher PEEP is associated with a higher risk of cardiovascular complications; the analysed ventilation strategies showed no differences in 30-day mortality.

The increase in the rate of PPCs is explained by changes in lung physiology during general anaesthesia.^32^, ^33^, ^34^ Muscle paralysis and patient position can reduce functional residual capacity and expiratory flow, resulting in atelectasis that can adversely impact thoracopulmonary mechanics and gas exchange.^35^ Moreover, high pressures, HVs, and cyclic opening of respiratory units can cause alveolar and endothelial dysfunction, leading to vessel leakage and inflammation, responsible for ventilator-induced lung injury.^7^ On the contrary, concurrent use of LVs and moderate levels of PEEP prevents atelectasis, volutrauma, and barotrauma. According to other studies, the use of LV strategy increases the homogeneity of the distribution of the tV in the lungs of the surgical patient.^32^ Our meta-analysis is in line with the results of Serpa Neto and colleagues^36^ who conducted an individual patient data meta-analysis; in fact, our findings highlighted that LV is associated with a low incidence of PPCs, particularly when PEEP is titrated to obtain the lowest driving pressure.

Even if the use of LVs is extensively supported by the literature, the optimal level of PEEP is still debated.^24^ Many studies adopting HP strategy concluded that PEEP improves dynamic compliance and maintains the physiologic end-expiratory lung volume. However, PROVHILO trial suggested that this strategy during open abdominal surgery does not protect against PPCs, and similar results were found in RCTs investigating the role of PEEP in laparoscopic surgery.^19^^,^^20^ PEEP is not the only factor affecting pulmonary outcome, and it should be set according to the level of driving pressure which has to be minimised, otherwise HP could result only in overstretching of the lung, without improvement in alveolar recruitment.^37^, ^38^, ^39^ Zhang and colleagues^30^ compared a fixed level of PEEP of 6 cm H_2_O with the incremental PEEP titration to achieve the lower driving pressure; the authors showed improvement of ventilation of dorsal dependent lung regions, contributing to a reduction of atelectasis and PPCs.

Our findings showed that PEEP level influences CVCs. In particular, the use of HP strategy increases the risk of hypotension in patients undergoing elective surgery; PEEP can influence venous return and both right and left ventricular function.^40^,^41^

We found that the mortality risk was not influenced by ventilation strategy. Our results are in accordance with the MECANO trial, which found no statistical differences in mortality rate after cardiopulmonary bypass between 756 patients assigned to a protective ventilation strategy group (tV 6 ml kg^−1^ of ideal body weight and PEEP of 5 cm H_2_O) and 745 patients assigned to a no ventilation strategy group.^42^ We found that mortality risk was similar for the DP group and the FP group, and no statistical differences were present in the LP or HP settings.

Our meta-analysis has some limitations: we included only articles in English and the enrolled studies had heterogenous inclusion and exclusion criteria. Moreover, we consider PEEP as a dichotomous variable, and it can considerably differ among HP *vs* LP groups. In addition, the definition and the time frame of PPCs are heterogenous across the studies, and minor and major complications are often mixed together. Many studies enrolled a low number of patients so there is the risk of a ‘small study’ effect, namely a bias raising from the tendency by small studies to publish and report larger and more advantageous effect size than studies with larger sample size. It is important to underline the potential risk of heterogeneity arising from the differences in other ventilatory parameters when only one of them is investigated as they are inter-related. In our analysis, the enrolled studies presented homogeneous values of ventilatory settings, including fraction of inspired oxygen (FiO_2_) and similar use of recruitment manoeuvres, but ventilatory frequency was not specified, even if its value can affect the incidence of PPCs.^43^ Another source of concern could be the different risk of PPCs; some authors assessed this risk using a validated score as ARISCAT. The composition of the study population in our meta-analysis is characterised by a high percentage of patients with intermediate-to-high risk for PPCs (i.e. 66–95%), particularly in the studies assessing the role of PEEP. Our meta-analysis excluded studies enrolling patients with severe pulmonary and cardiac disease so no conclusion can be drawn about this population of patients. Another source of heterogeneity is the definition of the duration of hypotension which is sometimes omitted. Moreover, it is important to keep in mind that hypotension is not synonymous with inadequate tissue perfusion, so it might not have any consequence. Many studies investigated levels of PEEP which are not representative of common settings in the operating theatre, suggesting that more studies should investigate the grey zone of moderate level of PEEP^11^^,^^19^^,^^20^^,^^25^^,^^28^^,^^29^^,^^44^ Another limitation is the low robustness of the majority of the studies, as shown by fragility index analysis.^31^ Finally, studies investigating the association between different ventilation strategies and postoperative complications rarely analyse their consequences on the quality of life of patients, which is a fundamental outcome in anaesthesia and intensive care research.^45^

In conclusion, our meta-analysis suggests that low tidal volume and low driving pressure-guided PEEP ventilation strategies could help reduce the incidence of postoperative pulmonary complications. Optimal intraoperative mechanical ventilation is not fully elucidated, and further studies are needed to investigate the influence of different ventilation strategies on postoperative pulmonary complications, cardiovascular complications, and mortality.

## Authors’ contributions

Conceived the work: PB

Assessed the risk of bias: PB, AUdS

Statistical analysis: CI

Critical revision of the manuscript: MV

Wrote the original manuscript draft: AC

Assessed the eligibility of the studies: FS, SN

Prepared tables and figures: AUdS

Supervised the work: GS

Wrote the final version of the manuscript: AM

## Declaration of interest

The authors declare that they have no conflicts of interest.
