# Supplementary material for: Impact of ventilation strategies on pulmonary and cardiovascular complications in patients undergoing general anaesthesia for elective surgery: a systematic review and meta-analysis
Source: Br J Anaesth. 2023 Oct 14;131(6):1093–101. doi: 10.1016/j.bja.2023.09.011 (PMC10687618; doi:10.1016/j.bja.2023.09.011)
Supplement: Multimedia component 1 [file mmc1.docx]

| **Author** | **Year** | **Surgery** | **Sample size** | **Population** | **Groups** | **Outcome** | **Results** |
| --- | --- | --- | --- | --- | --- | --- | --- |
| **Chun EH** | 2019 | Robotic gynecologic surgery | 40 | Female, 20 to 60 years, ASA 1-2.  Excluded for: current respiratory infection; COPD; glaucoma and BMI > 35. | High PEEP (8 cmH_2_O)  vs  Low PEEP (4 cmH_2_O) | Primary: oxygenation and respiratory mechanics.  Secondary: changes in ventilation distribution across the lung regions; PPCs. | The PIP and mean PAW of the low PEEP group were lower than those of the high PEEP group; the oxygenation factor in the low PEEP group was higher than that in the high PEEP group during mechanical ventilation at all times.  There was no difference in PPCs and in the fractional distribution of end-expiratory ventilation across the lung regions between the two groups. |
| **Futier E** | 2013 | Abdominal | 400 | Adults > 40 years old, scheduled for laparoscopic or non-laparoscopic elective major abdominal surgery, at intermediate to high risk of pulmonary complications.  Excluded for: recent mechanical ventilation; BMI ≥ 35 kg/m^2^; history of respiratory failure, recent sepsis or emergency surgery; progressive neuromuscular illness. | High tidal volume ventilation (tV 10-12 ml/kg of PBW, ZEEP and no RM)  vs  Low tidal volume ventilation (tV 8 ml/kg of PBW, PEEP of 6-8 cmH_2_O + RM) | Primary: major pulmonary and extrapulmonary complications occurring within the first 7 days after surgery.  Secondary: incidence of PPCs within 30 days after surgery; postoperative gas exchange; unplanned ICU admission; extrapulmonary complications; LoS in ICU and hospital; 30-day mortality. | The use of a LPV strategy was associated with improved clinical outcomes (included lower incidence of PPCs) and reduced health care utilization. |
| **Jiang L** | 2021 | Neurosurgery | 90 | Traumatic brain injury patients.  Excluded for: history of mental or neurological disorders; severe cardiac, pulmonary, renal or hepatic diseases; recent stroke, myocardial infarction or major surgery. | High tidal volume ventilation (tV 10mL/kg, ZEEP, no RMs);  vs  Low tidal volume ventilation (tV 8 mL/kg + PEEP 5 cmH_2_O) without RMs.  vs  Low tidal volume ventilation (tV 8 mL/kg + PEEP 5 cmH_2_O) + RMs. | Primary: incidence of total PPCs.  Secondary: intraoperative respiratory mechanics parameters; serum levels of brain injury markers; incidence of each postoperative pulmonary and neurological complications. | Protective ventilation groups had decreased 30-day incidence of PPCs, improved intraoperative PaO2 and dynamic compliance, decreased PIP and PAW; intraoperative hypotension was more frequent in the RM group. The postoperative incidences of neurological complications among the three groups were comparable. |
| **Longhini F** | 2021 | Neurosurgery | 60 | Adult patients undergoing major cerebral or spinal neurosurgical interventions with risk index for pulmonary postoperative complications > 2 and not expected to need postoperative ICU admission.  Excluded for: recent mechanical ventilation; BMI ≥ 35 kg/m^2^; history of respiratory failure, recent sepsis or emergency surgery; progressive neuromuscular illness. | Low tidal volume ventilation (tV 6 ml/kg of IBW, RR 16/min PEEP 5 cmH2O and RM)  vs  High tidal volume ventilation (tV 10 ml/kg of IBW, RR 6–8/min, ZEEP and no RM). | Primary: intraoperative adverse events (hypoxemia, hypotension or hypertension, bradycardia or tachycardia); the level of cerebral tension at dura opening; the intraoperative control of PaCO2.  Secondary: rate of pulmonary and extrapulmonary complications; the number of unplanned ICU admission; LoS in ICU and hospital; mortality. | No difference in the rate of intraoperative adverse events, postoperative pulmonary and extrapulmonary complications and major clinical outcomes, was found between groups. |
| **Mini G** | 2021 | Abdominal | 82 | Adult patient (aged 18-75 years) with ASA I or II, undergoing elective major open abdominal surgery (deﬁned by a duration of surgery > 2 h) under general anesthesia.  Excluded for: BMI <18 or >30 kg/m^2^; significant cardiac, pulmonary, renal or hepatic diseases; chest wall deformities; history of previous thoracic surgery. | Fixed PEEP (2 cmH2O until laparotomy and 5 cmH_2_O throughout the surgery)  vs  Titrated PEEP (10 minutes after laparotomy, PEEP was increased by 1 cmH_2_O incrementally from 2 cmH_2_O until lowest driving pressure was achieved) | Primary: atelectasis measured by LUS at the end of surgery (before and after extubation).  Secondary: respiratory mechanics; arterial blood gas; the requirement of perioperative rescue therapy to maintain SaO_2_ >95%; incidence of PPCs. | LUS were signiﬁcantly higher in the ﬁxed PEEP group both before and after extubation.  Incidence of PPCs were signiﬁcantly lower in the titrated PEEP group |
| **Park SJ** | 2016 | Abdominal | 62 | 18–70 aged patients with ASA I-II undergoing elective laparoscopic hepatobiliary surgery.  Excluded for: cardiopulmonary or hepatorenal disease, recent infections, recent ventilator support, previous thromboembolic disease or denial of informed consent. | High tidal volume ventilation with RM (tV 10 ml/kg)    vs  Low tidal volume ventilation (tV of 6 ml/kg with PEEP cmH_2_O). | Primary: the incidence of PPCs.  Secondary: incidence of postoperative desaturation; respiratory parameters; hemodynamic variables; LoS in hospital. | PPCs and desaturation occurred less frequently in protective ventilated group. No differences were observed in the LoS, arterial blood gas analysis and hemodynamic variables between the two groups. |
| **Pi X** | 2015 | Abdominal open | 63 | Adult patient of at least 60 years old undergoing non-laparoscopic abdominal elective major surgery.  Excluded for: BMI > 35; recent acute lung injury or respiratory distress; recent mechanical ventilation; hemodynamic instability; intractable shock; neuromuscular disease. | High volume (9 ml/kg IBW) and ZEEP  vs  Low volume (7 ml/kg IBW) and PEEP 8 cmH_2_O  vs  Low volume (7 ml/kg IBW), PEEP 8 cmH_2_O and RM. | Primary: Intraoperative PaO2/FiO2 ratio and pulmonary compliance  Secondary: PPCs; LoS in hospital; acute heart failure; in-hospital death. | There were no significant differences in intraoperative PaO_2_/FiO_2_ ratio and FEV1 or FVC among the three groups. On the first postoperative day, the low volume with high PEEP and RM group had significantly higher FEV1 and showed advantages in maintaining the pulmonary compliance.  There were no significant differences in secondary outcome. |
| **PROBESE** | 2019 | Non-cardiac and non-neurological | 1976 | Adults with BMI ≥ 35 kg/m^2^ and ARISCAT score ≥ 26, undergoing laparoscopic or non- laparoscopic surgery that need at least 2h of general anesthesia.  Excluded for: previous lung surgery or IMV during previous 2 months; intraoperative one-lung ventilation; planned reintubation after surgery; need for intraoperative prone or lateral decubitus positioning during surgery; severe cardiac, lung, neurological, and neuromuscular disease. | High PEEP 12 (cmH_2_O) + RM  vs  Low PEEP (4 cmH_2_O) | Primary: composite of PPCs within the first 5 postoperative days.  Secondary: composite of severe PPCs; postoperative extrapulmonary complications; impaired postoperative wound healing; unplanned ICU admission or readmission; number of hospital-free days at postoperative day 90; intraoperative hypoxemia; intraoperative hypotension; intraoperative bradycardia; in-hospital mortality. | High PEEP + RM vs low PEEP strategy did not reduce PPCs.  During the intraoperative period, hypoxemia was less common while hypotension and bradycardia were more frequent in the high PEEP group. |
| **PROVE** | 2014 | Abdominal open | 900 | Adult patients at intermediate or high risk of having PPCs.  Excluded for: BMI > 40 kg/m^2^; severe cardiac or pulmonary or other comorbidities. | High PEEP (12 cmH_2_O) with RM  vs  Low PEEP (≤2 cmH_2_O) without RM. | Primary: composite of PPCs occurring in the first 5 days.  Secondary: intraoperative desaturation or hypotension; massive transfusion; surgical complications; any postoperative extrapulmonary complications. | A strategy with a high PEEP and RM during open abdominal surgery does not protect against PPCs, developed intraoperative hypotension, and needed more vasoactive drugs. |
| **Talab HF** | 2009 | Laparoscopic bariatric | 66 | Adult obese patients with BMI between 30 and 50 kg/m^2^.  Excluded for: history of heart or lung diseases. | Each group received a RM with a CPAP of 40 cmH_2_O for 7-8 second after intubation, a tV of 8-10mL/kg of IBW and three different level of PEEP:  ZEEP  vs  PEEP 5 cmH_2_O  vs  PEEP 10 cmH_2_O | Primary: postoperative lung atelectasis valued with CT scan.  Secondary: heart rate, non-invasive mean arterial pressure, and arterial oxygen saturation, alveolar arterial PaO_2_ gradient, bronchospasm, chest infection, desaturation, barotraumas, PACU stay. | Patients in the PEEP 10 cmH_2_O group had better oxygenation both intraoperatively and postoperatively, lower atelectasis score on chest CT scan, shorter PACU stay, and less PPCs than the ZEEP and PEEP 5 groups. No significant differences in MAP and HR. |
| **Wei K** | 2018 | Laparoscopic sleeve gastrectomy | 36 | Adult patients with BMI >40 kg/m^2^, ASA 2 or 3, aged between 18 and 65 years.  Excluded for: pulmonary infection or active asthma; PaCO_2_>80 mmHg or PaO_2_<60 mmHg in preoperative ABG analysis; history of heart or lung diseases. | tV of 8 mL/kg of IBW, no RM and ZEEP  vs  RM + ZEEP  vs  RM + PEEP 8 cmH_2_O at the end of RMs and maintained till the end of the procedure. | Primary: the evaluation of the time between the anesthesia and extubation.  Secondary: PACU stay, PPCs, hospital stay, time to first solid food intake resumption, MAP, heart rate. | RM with or without PEEP during pneumoperitoneum may similarly improve the oxygenation of obese patients. RMs may contribute to lung protection by inducing a decreased driving pressure and tend to more stable hemodynamic without PEEP. Reduced tidal volume combined with high PEEP during intraoperative ventilation showed protective effects against PPCs. |
| **Weingarten TN** | 2010 | Abdominal open | 40 | Geriatric patient (age>65 years).  Excluded for: obstructive or restrictive pulmonary disease; active asthma; previous lung surgery; home oxygen therapy; significant cardiac dysfunction; BMI>35 kg/m^2^ | ‘Open lung’ strategy (tV 6 mL/ kg PBW, PEEP 12 cmH_2_O and RM)  vs  High tidal volume ventilation (tV 6 ml/ kg PBW, ZEEP and no RM) | Primary: oxygenation; respiratory system mechanics; haemodynamic stability.   Secondary: serum levels of IL-6 and IL-8 before and after surgery; PPCs; duration of postoperative oxygen use; patients’ mental status during the ﬁrst 24 postoperative hours; death. | The number of postoperative days of supplemental oxygen, PPCs, length of hospital stay, and hemodynamic stability were not different between the groups. The recruitment manoeuvre improved intraoperative PaO2 and PaCO2 mildly. After the RM dynamic compliance increased on average 36% and airway resistance decreased on average 21%. The increase of IL-6 and IL-8 after surgery did not differ between the two groups. |
| **Wetterslev J** | 2001 | Abdominal | 38 | Adult patient of 29–77 years old, ASA I or II, and scheduled for upper abdominal surgery.  Excluded for: history of cardiopulmonary disease and BMI > 35 kg/m^2^. | PEEP titrated to achieve the highest pulmonary compliance  Vs  ZEEP | Primary: PaO_2,_ SpO_2_, PPCs.  Secondary: VAS score. | Perioperative PaO2 improved in PEEP group, but no significant differences were found in the four postoperative days. There was no difference in incidence of postoperative complications; the incidence of postoperative pneumonia in the ZEEP group was 32% and 10% in the PEEP group with a difference of 22%. |
| **Whalen FX** | 2006 | Bariatric laparoscopic | 20 | Adult obese patient between 25 and 65 years, ASA II or III with BMI>40 kg/m^2^  Excluded for: severe pulmonary disease; active asthma; previous lung surgery; home oxygen therapy. | tV of 8 mL/kg IBW, PEEP 4 cmH_2_O  vs  tV 8 mL/kg IBW with increasing PEEP in a stepwise fashion-first to 10 cmH_2_O (3 breaths), then to 15 cmH_2_O, until a peak of 50 cmH_2_O. | Primary: Pao2/FiO_2_.  Secondary: oxygenation, ventilation, respiratory mechanics, and hemodynamic. | The recruitment increased only intraoperative PaO_2_ and temporarily increased respiratory system dynamic compliance.  Mean arterial blood pressure and heart rate were not different between the two groups. Patients in the recruitment group received a larger total dose of vasopressors. No differences between the arterial and etCO2 were observed between groups. The length of hospitalization and incidence of PPCs were not significantly different. |
| **Xu Q** | 2022 | Laparoscopic Abdominal | 51 | Patients older than 65 years who were scheduled to undergo elective laparoscopic surgery of expected duration greater than 2 h, with ASA II and III and a BMI <30 kg/m^2^.  Excluded for: history of severe lung disease or thoracic surgery, preoperative renal replacement therapy, and congestive heart failure (NYHA III or IV) | Low tidal volume ventilation with individualized PEEP guided by ΔP (until the lowest ΔP was reached)  vs  Low tidal volume ventilation with standardized PEEP 6 cmH_2_O  vs  High tidal volume ventilation (tV 10ml/kg IBW without PEEP or RM) | Primary: LUS noted at the end of surgery and 15 minutes after admission to the PACU.  Secondary: perioperative oxygenation function, expiratory mechanics, and the incidence of the PPCs. | The LUS, the lung static compliance (C_stat_) and ΔP of the individualized PEEP group were significantly better.  Intraoperative titrated PEEP reduced PPCs and improved respiratory mechanics; meanwhile, standard PEEP strategy is not superior to high tidal volume ventilation. |
| **Zhang C** | 2021 | Open Abdominal | 148 | 18-80 years aged patients scheduled for open upper abdominal surgery that would last for at least 2h under general anesthesia, with intermediate-to-high ARISCAT score, ASA II and III and BMI < 35kg/m^2^.  Excluded for: recent history of upper respiratory tract or pulmonary infection, serious cardiovascular, lung, kidney, or hematopoietic diseases. | Individualized PEEP guided by minimum driving pressure (from 0 to 14 cmH_2_O)  vs  Fixed PEEP of 6 cmH_2_O. | Primary: incidence of PPCs within the first 7 days after surgery.  Secondary: the severity of PPCs, ICU admission rate, LoS in hospital, and 30-day mortality | The incidence of clinically significant PPCs (grade 2+) was significantly lower in the individualized PEEP group than that in the fixed PEEP group. The median value of PEEP in the individualized group was 10 cm H2O.  The severity score of PPCs was significantly reduced in the individualized PEEP group compared with that in the fixed PEEP group. Higher respiratory compliance during surgery and improved intra- and postoperative oxygenation was observed in the individualized group. No significant differences were found in other outcomes. |

Supplemental Table 1. Characteristics of the enrolled studies. BMI= Body Mass Index; ARISCAT= The Assess Respiratory Risk in Surgical Patients in Catalonia score estimates the risk of postoperative pulmonary complications, with scores greater or equal than 45 indicating high risk; PEEP= Post-end expiratory pressure; RM= Recruitment Maneuver; tV= Tidal Volume; PBW= Predicted Body Weight; PPCs= Post-operative Pulmonary Complications; ICU= Intensive Care Unit; ASA= American Society of Anesthesiologists; COPD= Chronic Obstructive Pulmonary Disease; LUS= Lung Ultrasound Score; PIP= Peak Inspiratory Pressure; PAW= Pressure Airway; CABG= Coronary Artery Bypass Graft surgery; ZEEP= Zero-End Expiratory Pressure; ECC= Emergency Cardiac Care; LoS= Length of Stay; AKI= Acute Kidney Injury; CPB= Cardiopulmonary Bypass; LPV= Lung Protective Ventilation; MAP= Mean Arterial Pressure; HR= Heart rate; FEV1= forced expiratory volume; FVC= Functional Vital Capacity; PACU= Post-Anesthetic Care Unit; VCV= Volume Controlled Ventilation; VAS score= Visual Analogue Scale score; DP= Driving Pressure.

| Author | YEAR | LVvs HV | | | HP vs LP | | | DP vs FP | | |
| --- | --- | --- | --- | --- | --- | --- | --- | --- | --- | --- |
|  |  | PPCs | CVCs | Death | PPCs | CVCs | Death | PPCs | CVCs | Death |
| **Wetterslev J** | 2001 | - | - | - | - | - | - | 0 | 0 | 0 |
| **Whalen FX** | 2006 | - | - | - | 0 | 0 | 0 | - | - | 0 |
| **Talab HF** | 2009 | 0 | 0 | 0 | - | - | - | - | - | - |
| **Weingarten TN** | 2010 | - | 0 | 0 | - | - | - | - | 0 | - |
| **Futier E** | 2013 | - | - | - | 0 | 23 | 0 | - | - | - |
| **PROVE** | 2014 | 0 | 0 | 0 | - | - | - | - | - | - |
| **Pi X** | 2015 | 0 | - | 0 | - | - | - | - | - | - |
| **Park SJ** | 2016 | - | - | - | 0 | 1 | 0 | - | - | - |
| **Wei K** | 2018 | 0 | 0 | 0 | - | - | - | - | - | - |
| **PROBESE** | 2019 | - | - | - | 0 | 8 | 1 | - | - | - |
| **Chun EH** | 2019 | - | - | - | 0 | - | - | - | - | - |
| **Jiang L** | 2021 | 0 | - | 0 | - | - | - | - | - | - |
| **Longhini F** | 2021 | 0 | 0 | 0 | - | - | - | - | - | - |
| **Mini G** | 2021 | - | - | - | - | - | - | - | - | 0 |
| **Zhang C** | 2021 | - | - | - | - | - | - | 0 | 4 | - |
| **Xu Q** | 2022 | - | 0 | 0 | - | 0 | 0 | - | 0 | 0 |
|  | **Median (min;max)** | 0 (0;0) | 0 (0;0) | 0 (0;0) | 0 (0;0) | 1 (0;23) | 0 (0;1) | 0 (0;0) | 0 (0;4) | 0 (0;0) |

Supplemental Table 2. Fragility indexes, median, minimum and maximum of the enrolled studies. LV= low tidal volume; HP= high tidal volume; PPCs= Post-operative Pulmonary Complications; CVCs= Cardio-vascular Complications; HP= High PEEP; LP= Low PEEP; DP= Driving Pressure; FP= Fixed PEEP. The fragility index indicates the number of patients which should have experienced a different outcome to turn a statistically significant result in a non-significant one: the smaller this number is, the less robust is the conclusion of the study because it means that it is based on a little difference between patients in the “event” and “no event” groups.


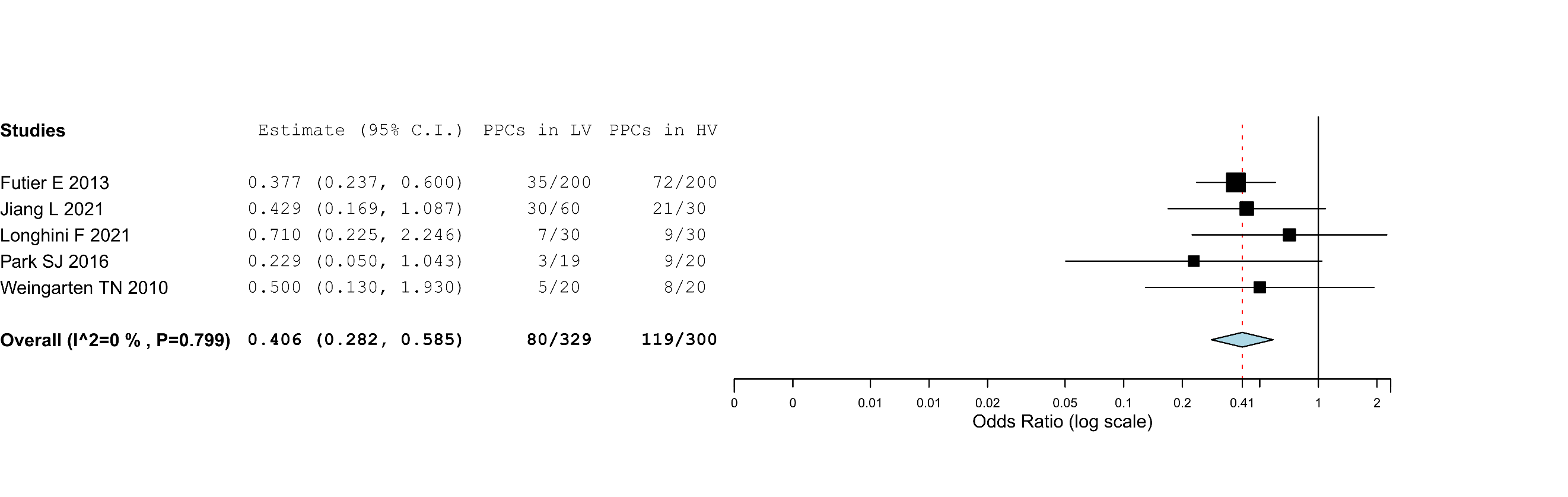

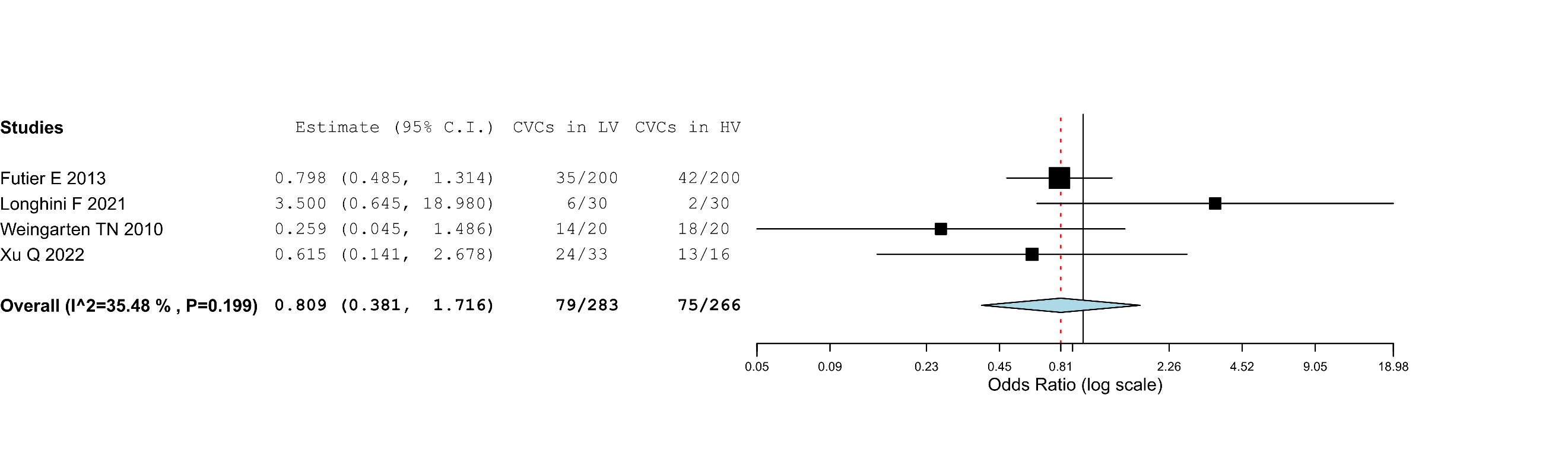

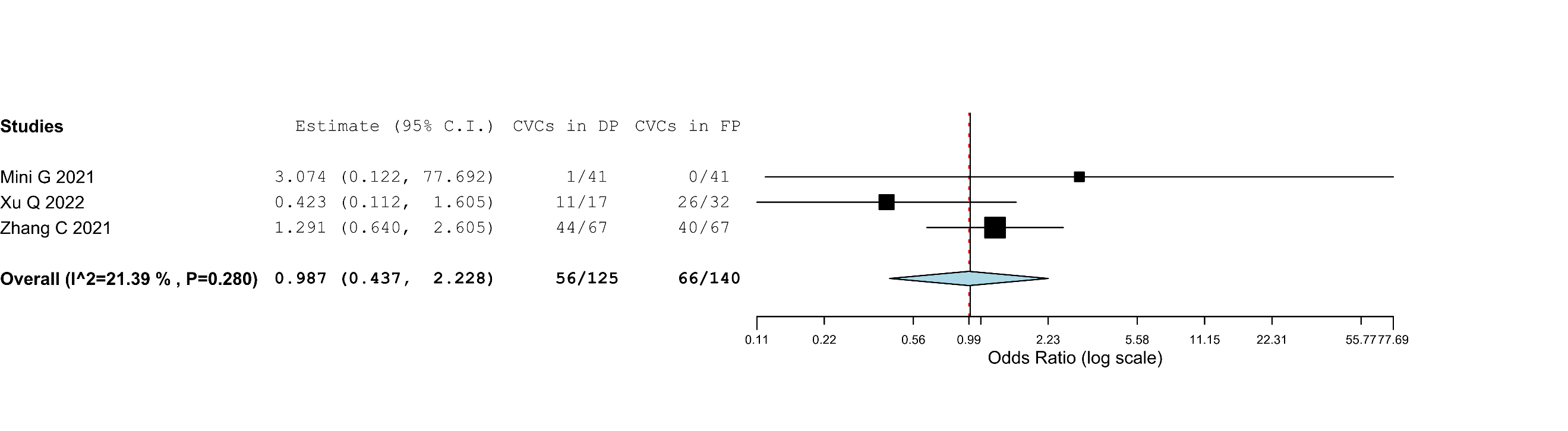

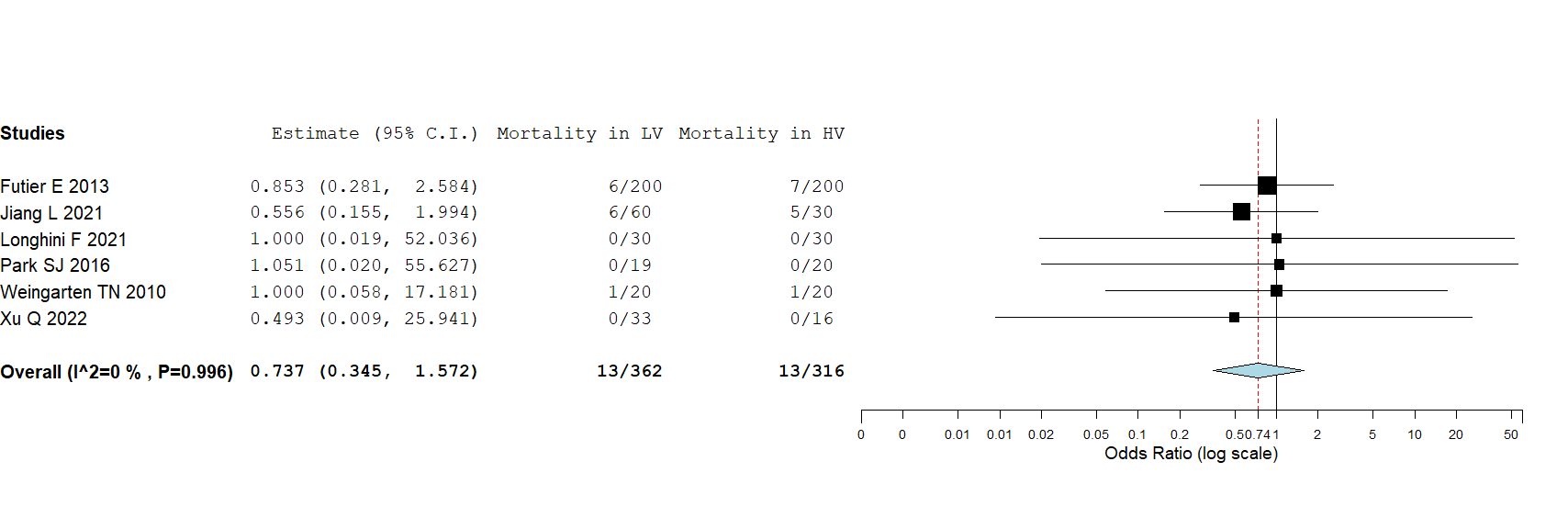

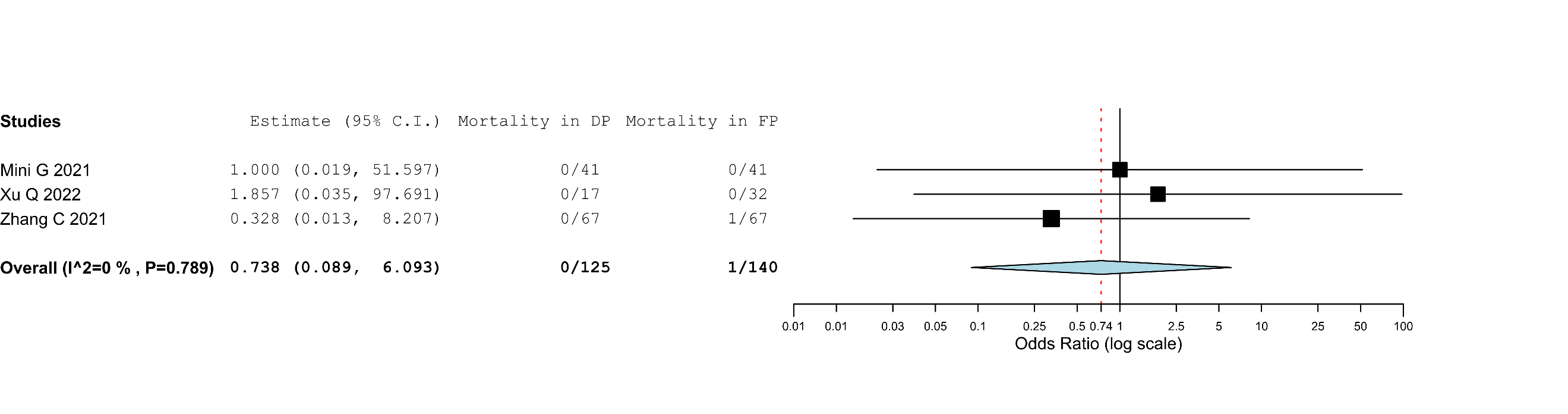


**c**

**e**

**d**

**b**

**a**

Supplemental Figure 1. Sensitivity analysis conducted excluding studies with ‘high’ risk of bias: a) Postoperative pulmonary complications (PPCs) between low tidal volume (LV) ventilation and high tidal volume (HV) ventilation strategies; b) Cardiovascular complications (CVCs) between LV and HV strategies; c) CVCs between driving pressure guided post-expiratory end pressure (DP) and fixed post-expiratory end pressure (FP) strategies; d) Mortality between LV ventilation and HV ventilation strategies; e) Mortality between DP and FP.


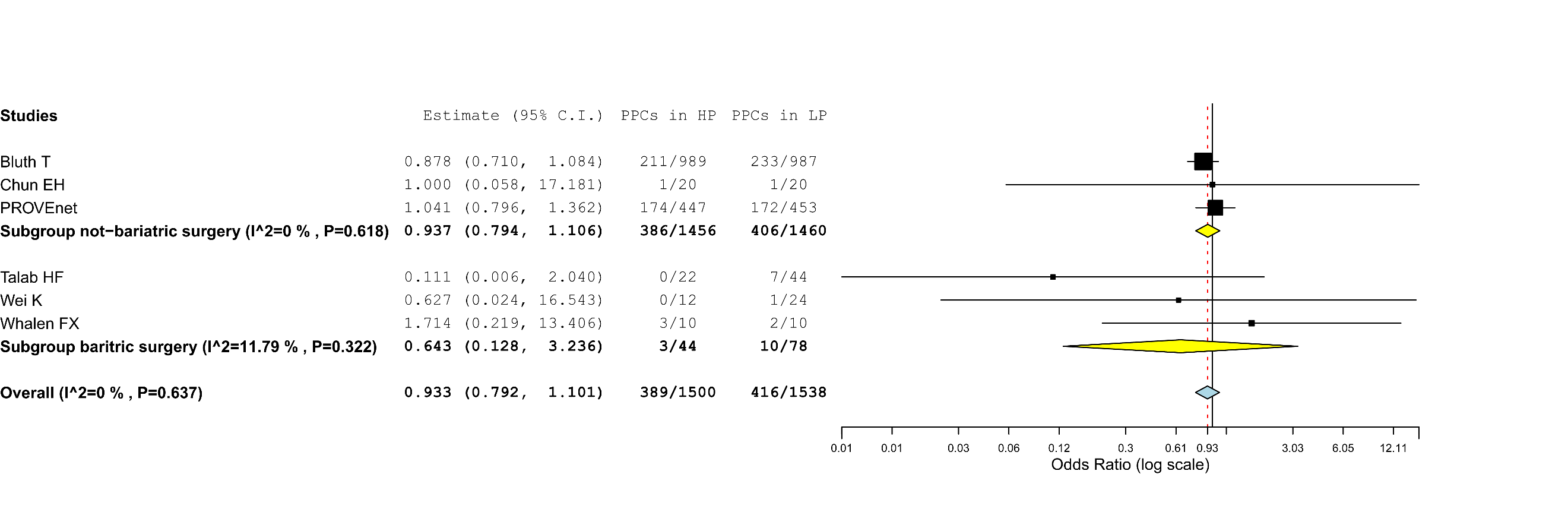

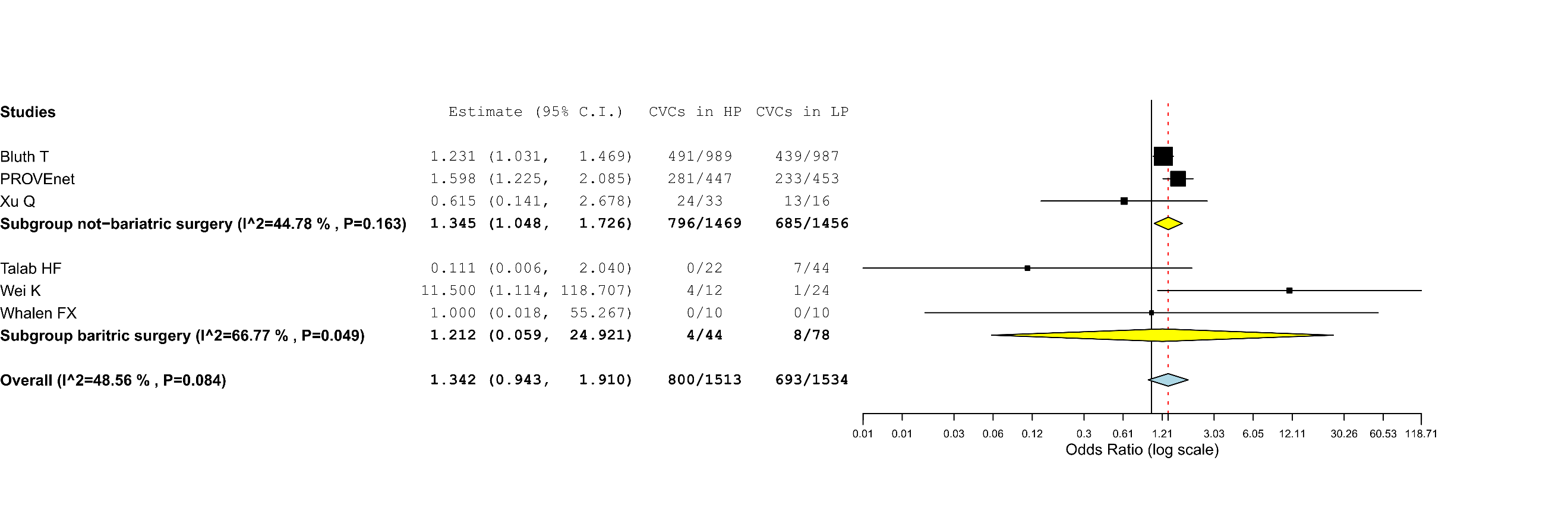

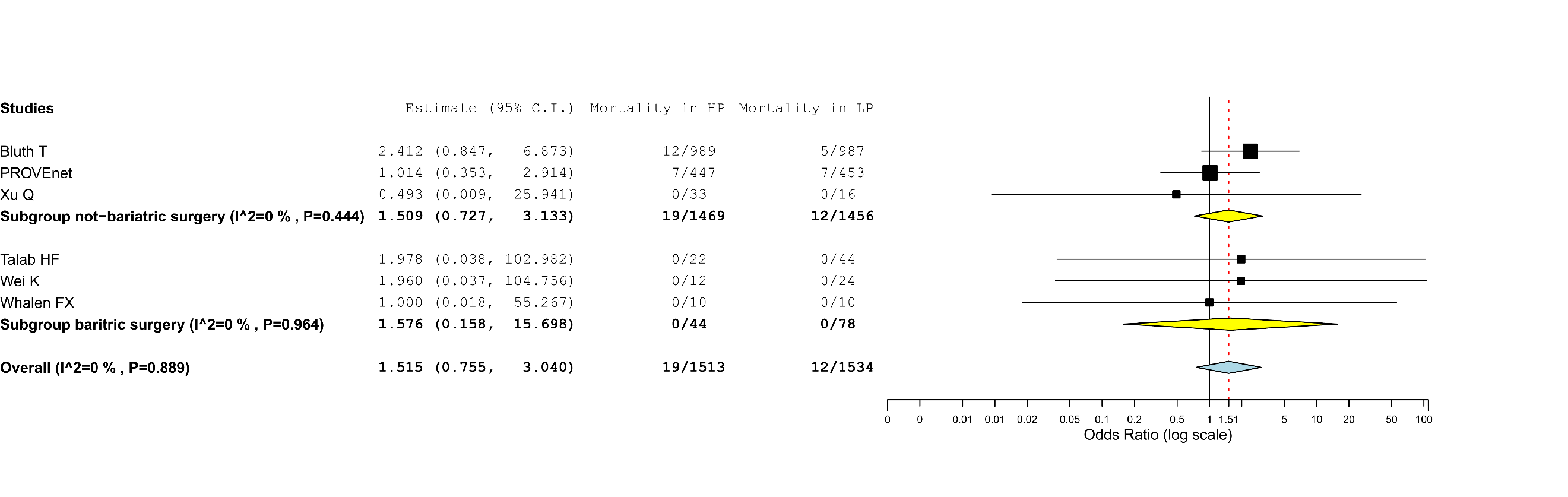


**c**

**e**

**b**

**a**

Supplemental Figure 2. Analysis of bariatric and not-bariatric surgery subgroups: a) Postoperative pulmonary complications between high post-expiratory end pressure (HP) and low post-expiratory end pressure (LP); b) Cardiovascular between HP and LP strategies; c) Mortality between HP and LP strategies.


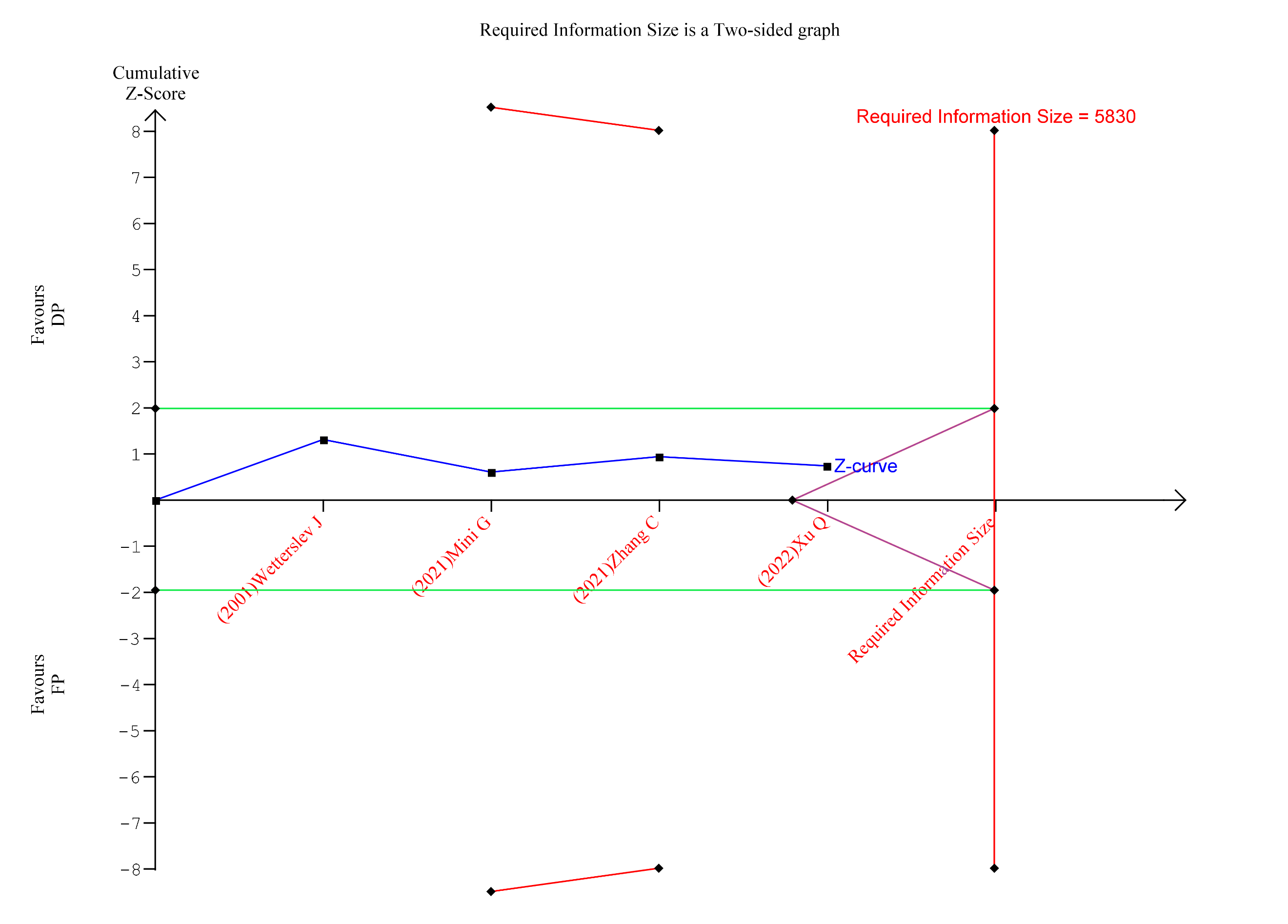

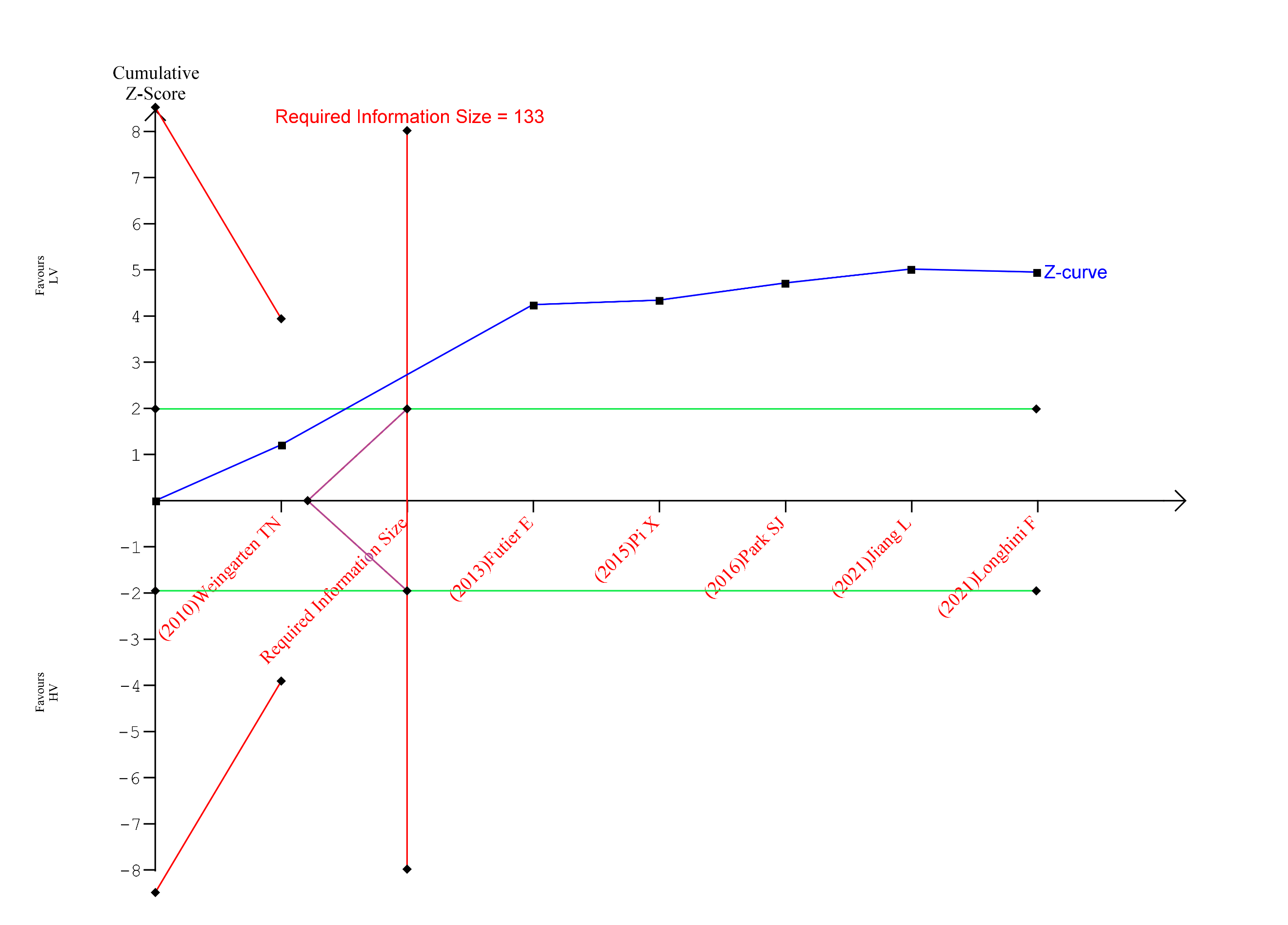

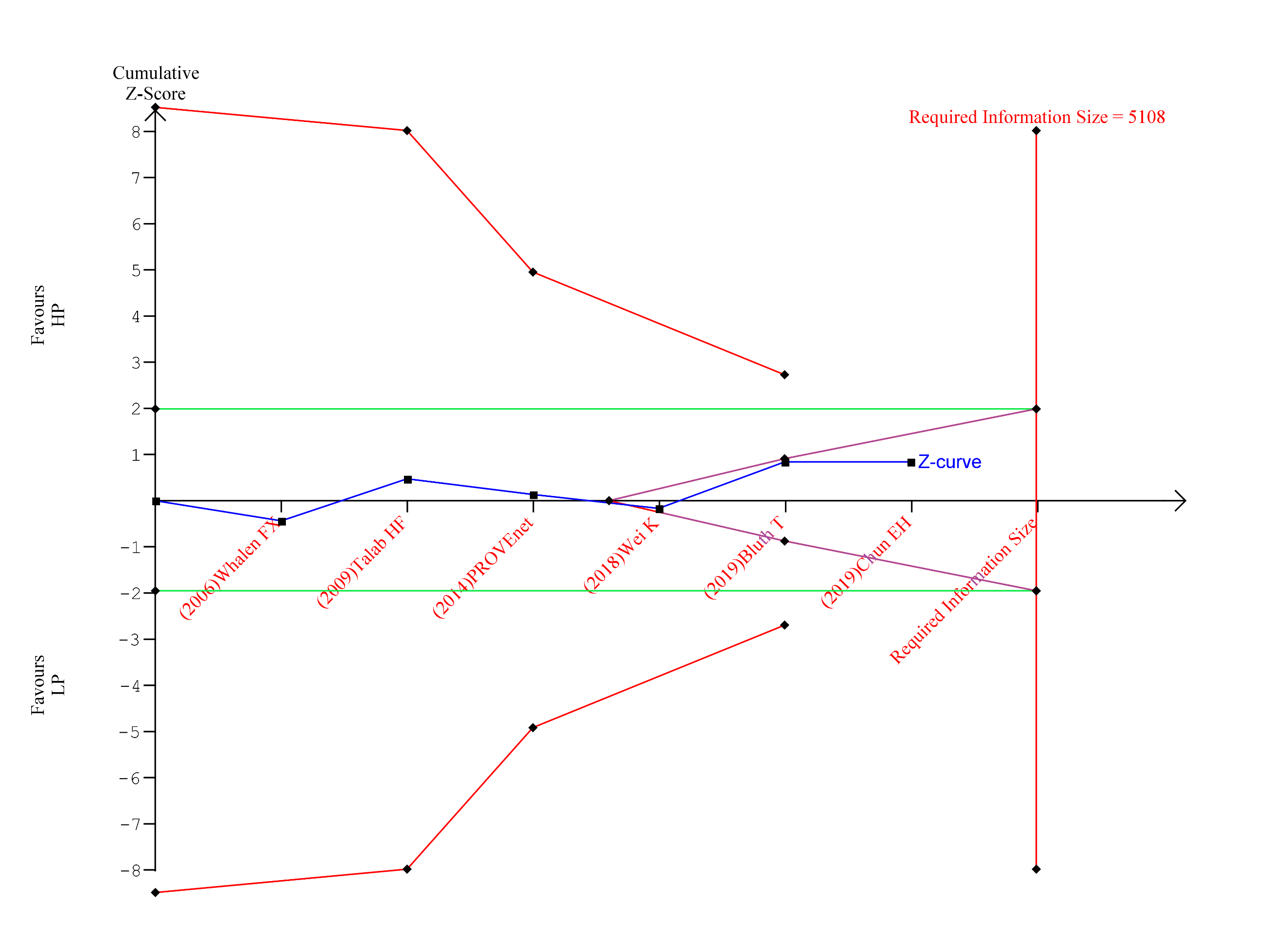

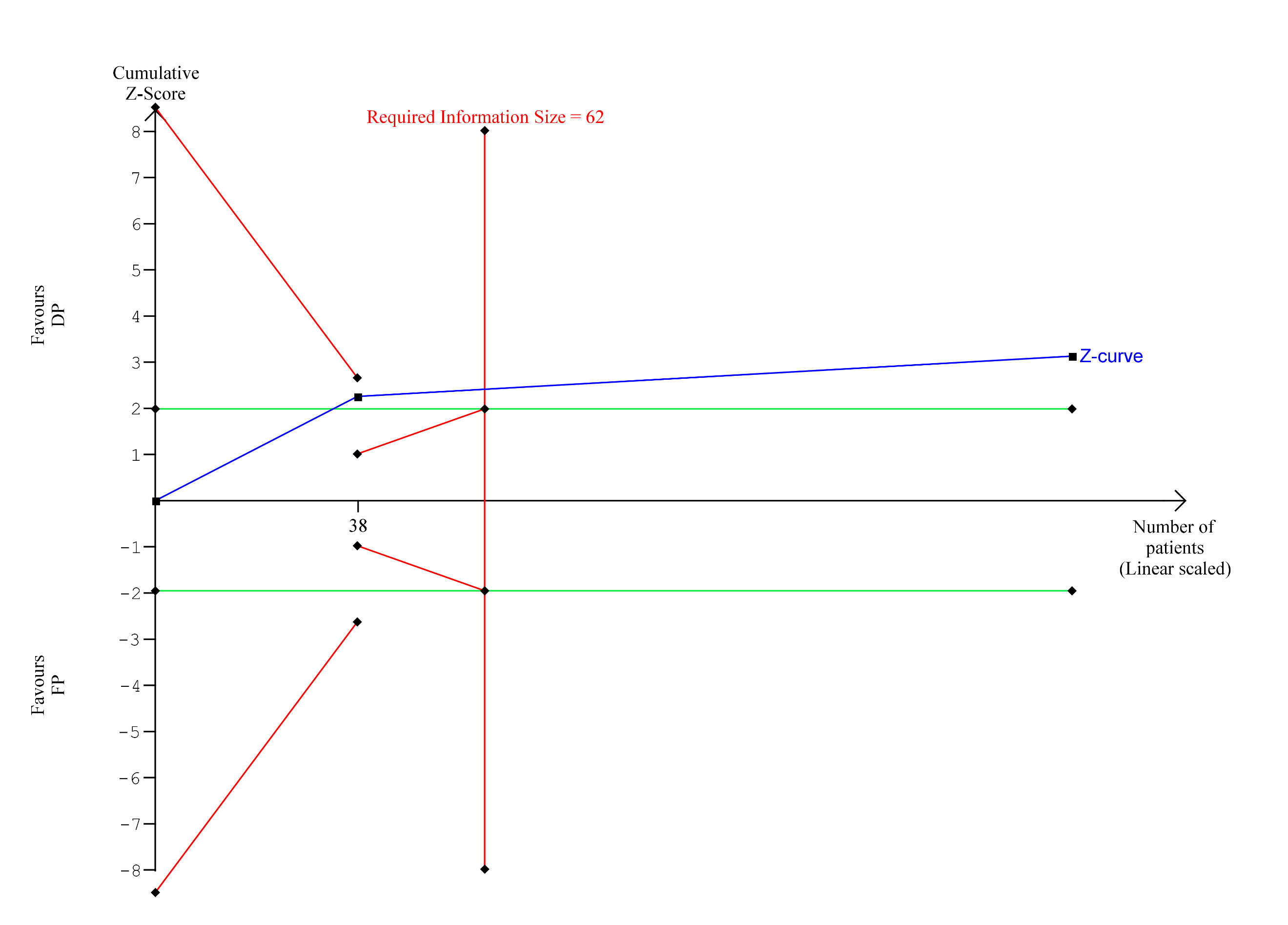

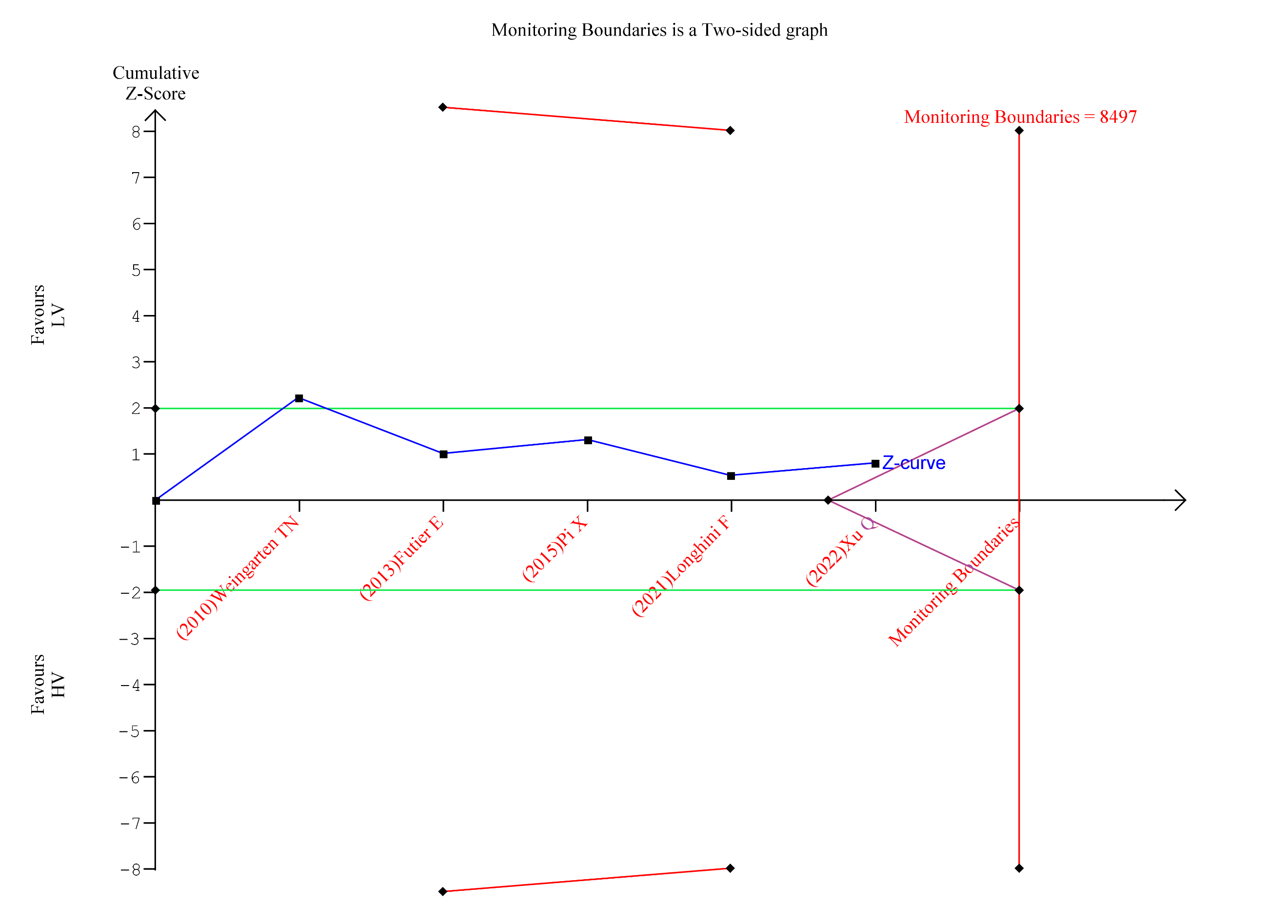

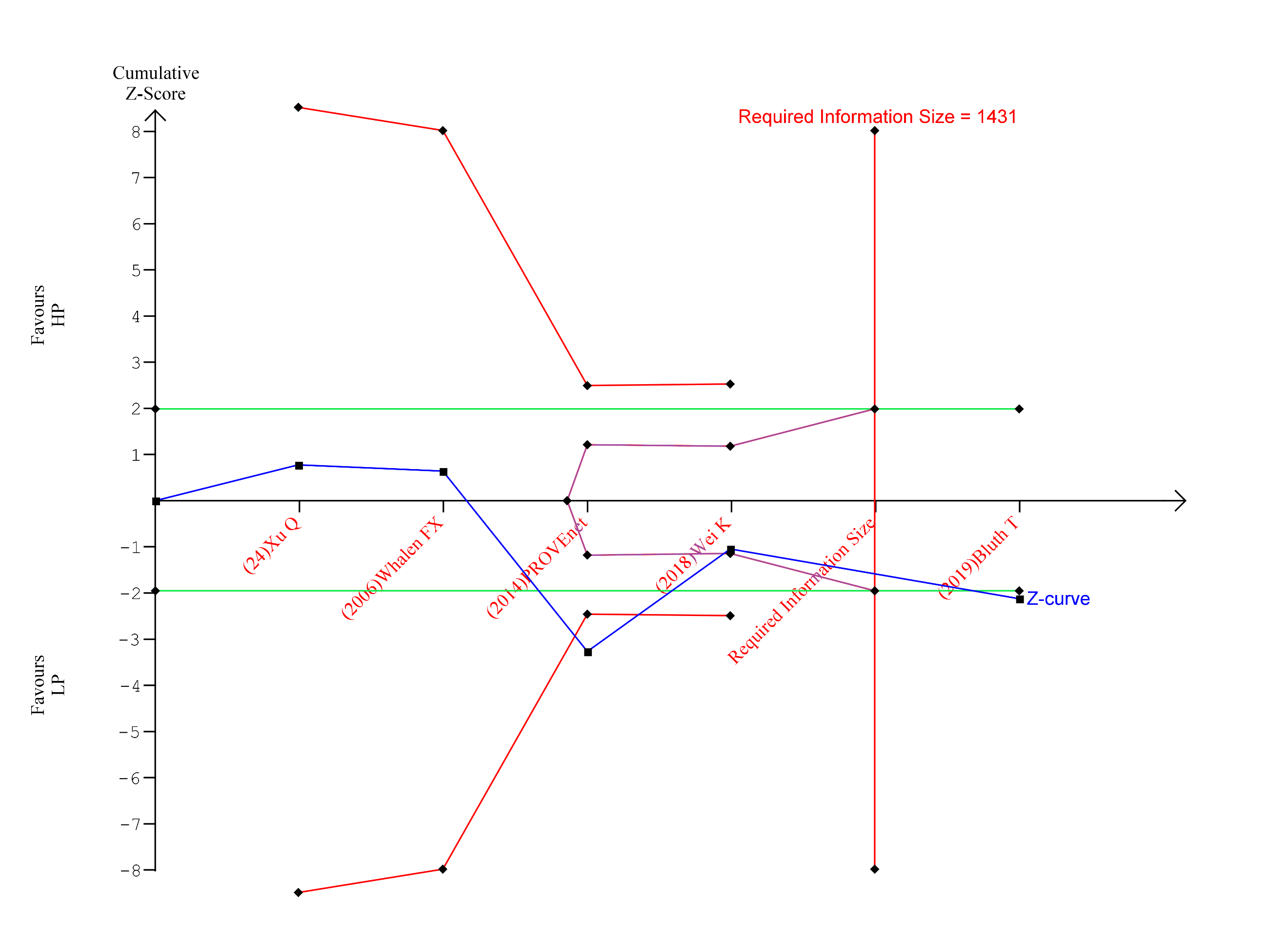

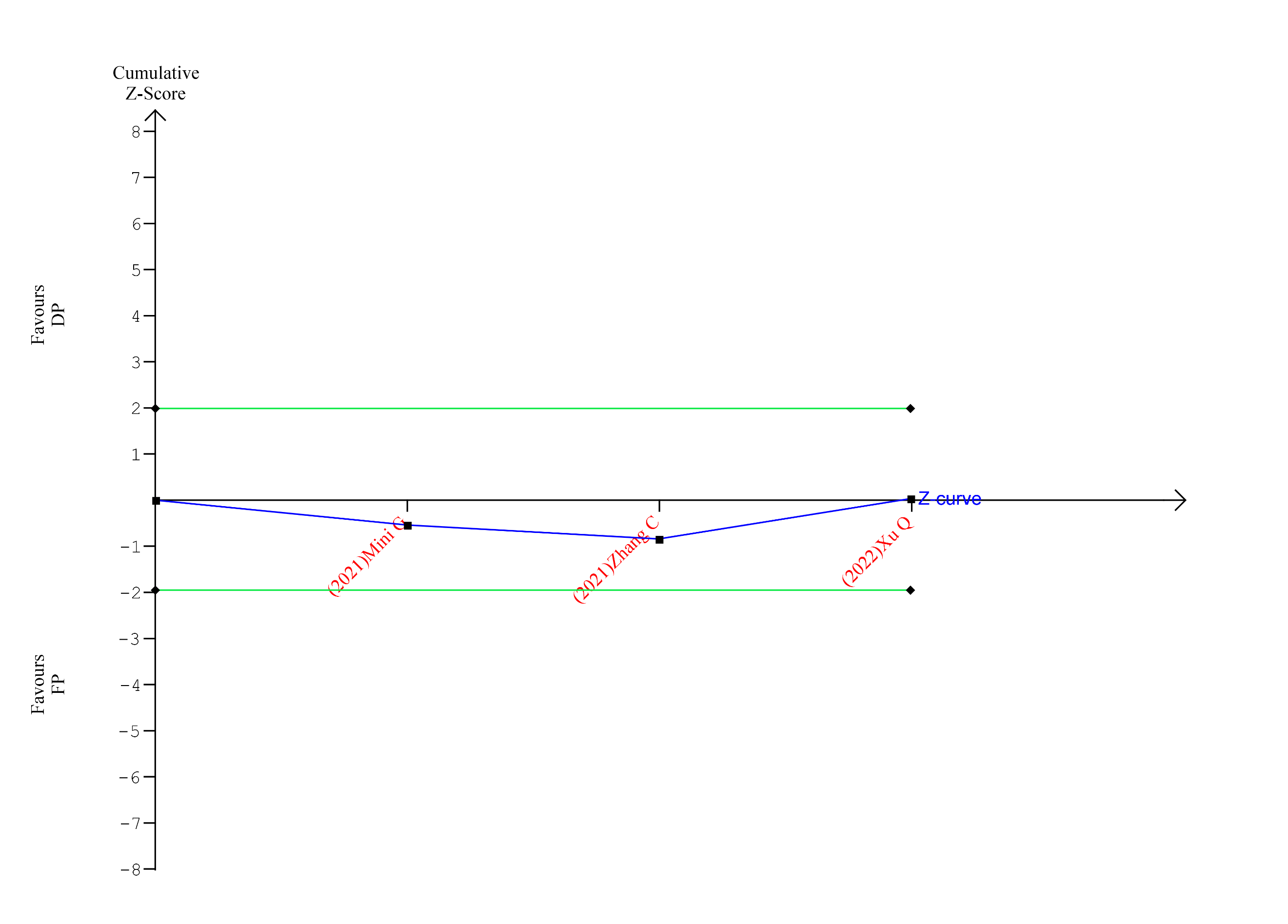

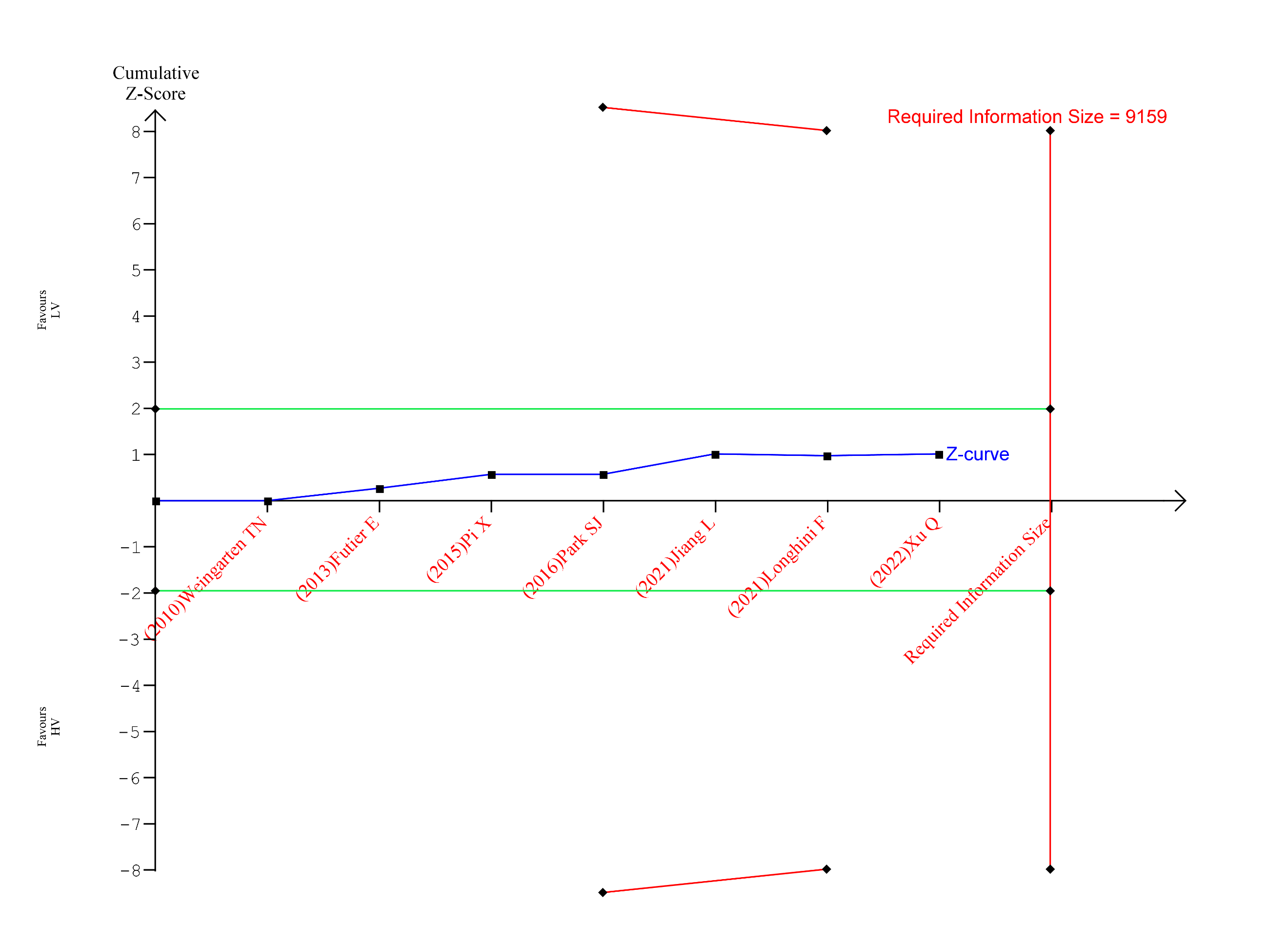

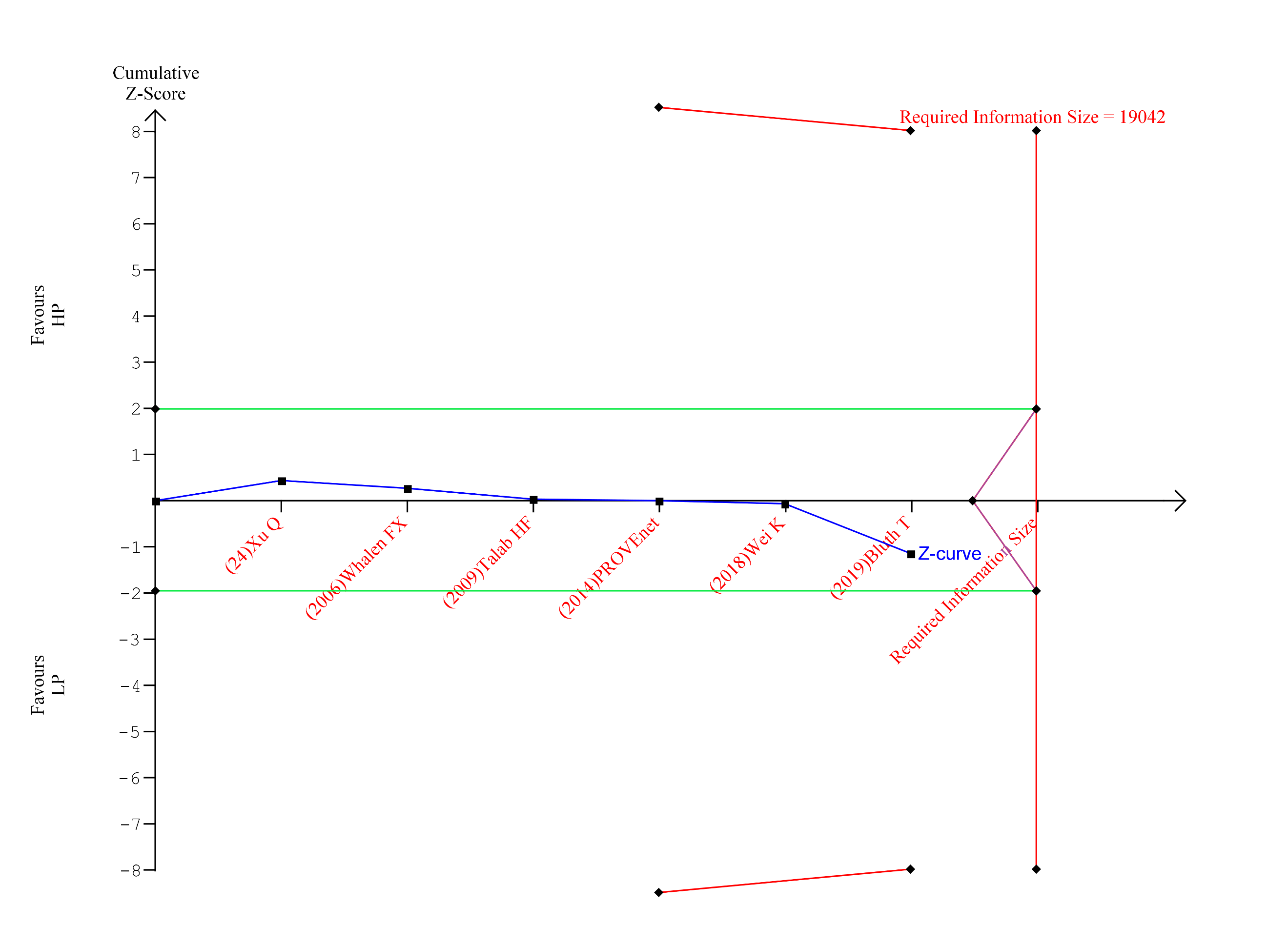


**e**

**i**

**g**

**h**

**f**

**d**

**c**

**a**

**b**

Supplemental Figure 3. Trial sequential analysis (TSA) of the different outcomes between the strategies: a) Postoperative pulmonary complications (PPCs) between low tidal volume (LV) ventilation and high tidal volume (HV) ventilation strategies; b) PPCs between high post-expiratory end pressure (HP) and low post-expiratory end pressure (LP) strategies ; c) PPCs between driving pressure guided post-expiratory end pressure (DP) and fixed post-expiratory end pressure (FP) strategies; d) cardiovascular complications (CVCs) between LV ventilation and HV ventilation strategies; e) CVCs between HP and LP strategies; f) CVCs between DP and FP strategies; g) mortality between LV ventilation and HV ventilation strategies; h) mortality between HP and LP strategies; i) mortality between DP and FP strategies. Interpretation of TSA graphs: on the x-axis is reported the cumulative number of patients enrolled for each outcome for each study, while on y-axis is reported the cumulative z-score. The two horizontal red lines represents the usual z values to consider a result statistically significant (i.e., -1.96 and 1.96, corresponding to a p value=0.05). The descending and ascending red lines in the upper and lower part of the graph are the benefit and harm boundaries (monitoring boundaries), respectively, and they represent the cumulative z-score calculated by chronologically adding each study: if the blue line crosses one of these boundaries, the result can be considered statistically significant. The vertical red line represents the required information size. The required information size is defined as the number of participants and events necessary to detect or reject an a priori assumed intervention effect; it is calculated taking into account the heterogeneity of the enrolled studies: if the cumulative z-score line crosses the required information size, the meta-analysis can be considered conclusive for the selected outcome. The two diverging straight lines (in purple) starting from the x axis represent the futility boundaries: if the cumulative z-score crosses these lines, it is very unlikely that the meta-analysis could detect a statistically significant difference between groups even if other studies will be added to reach the required information size.


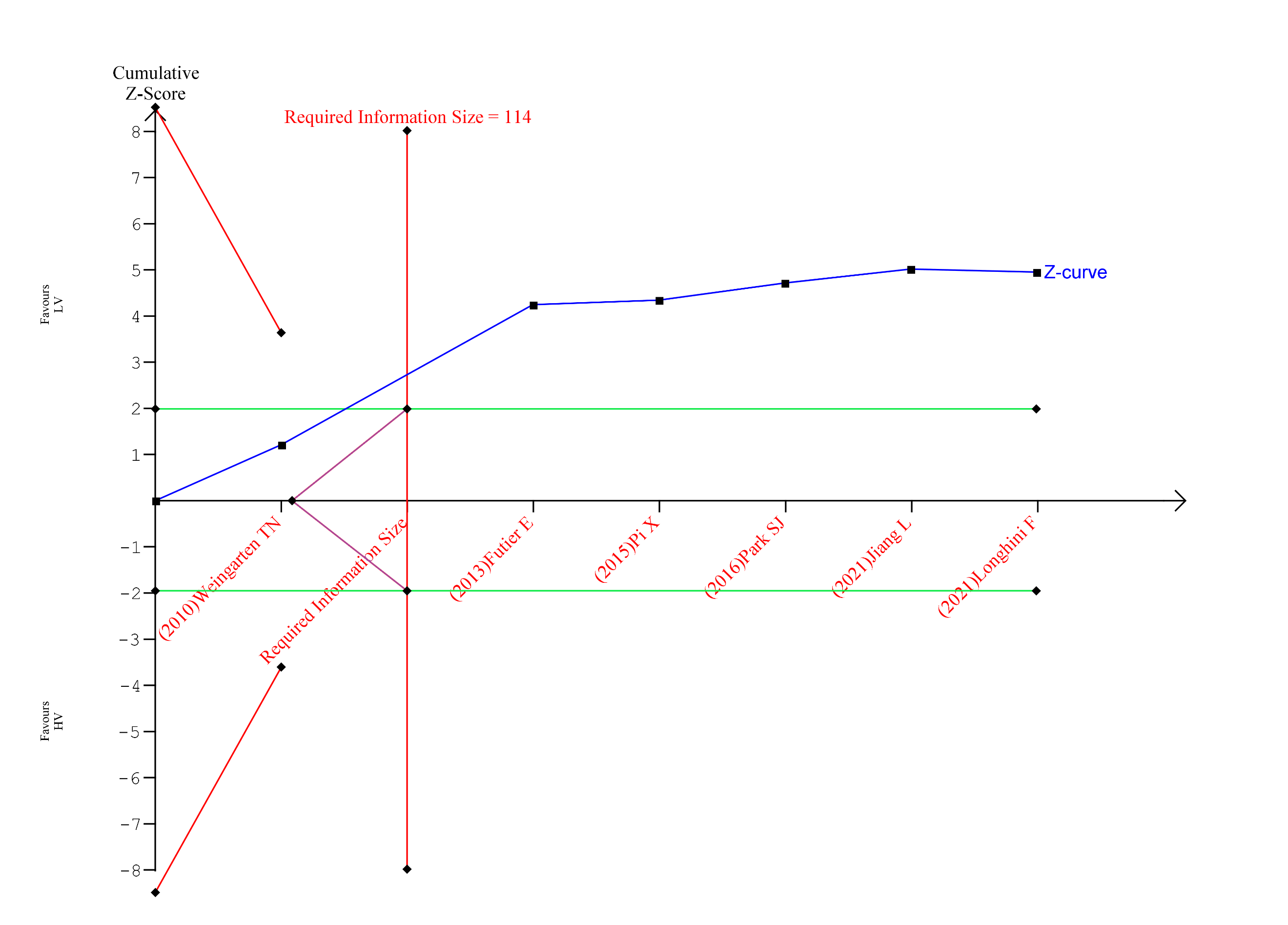

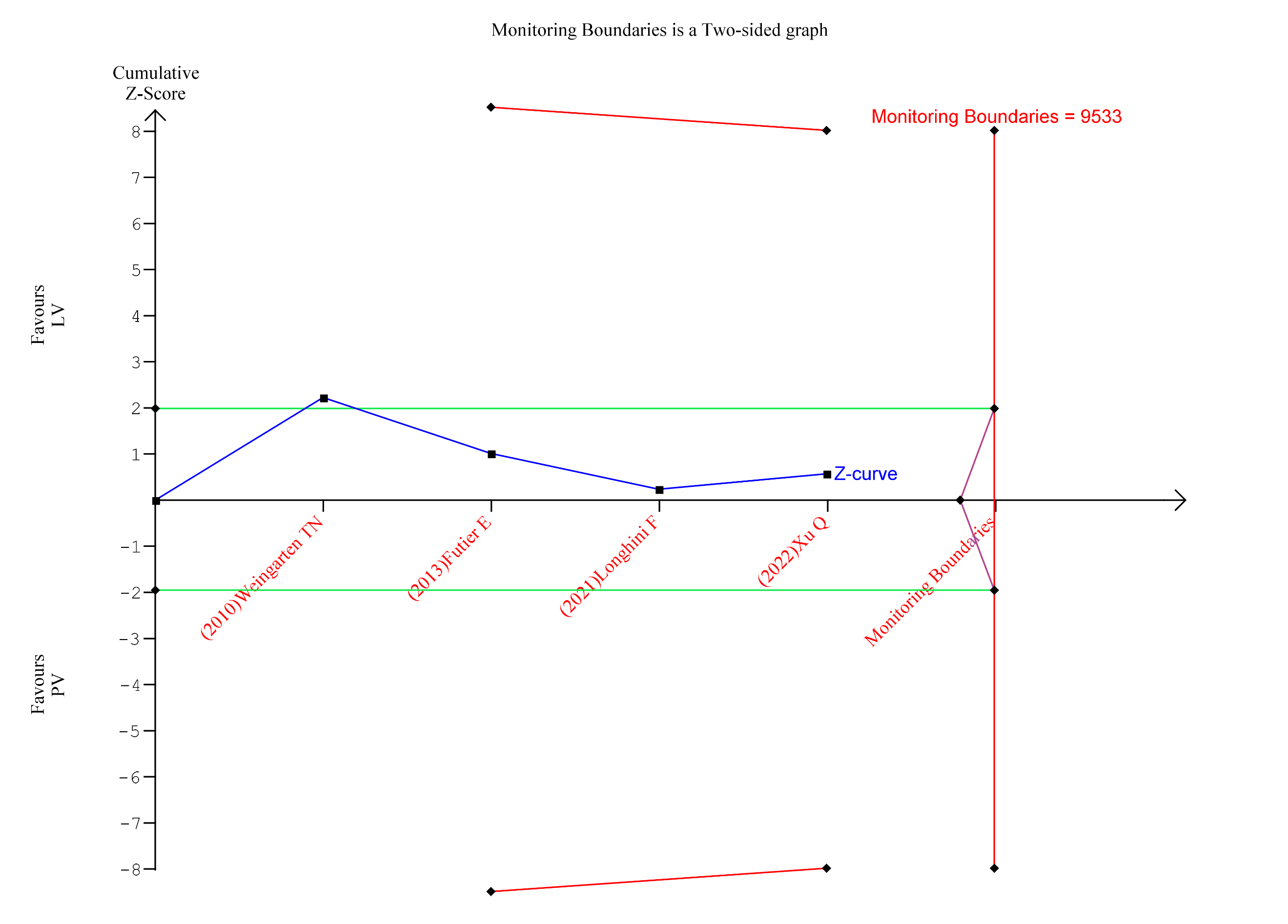

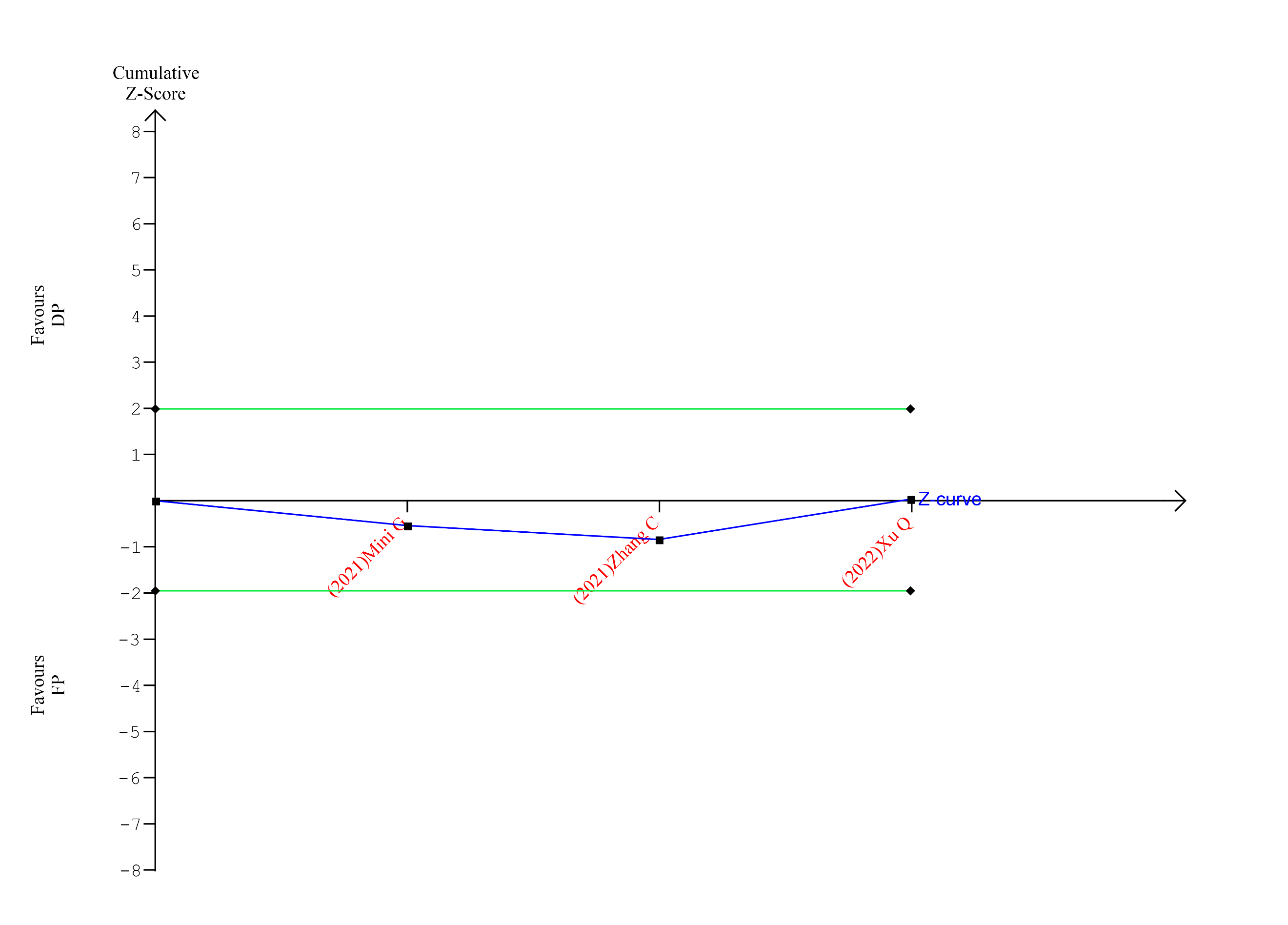

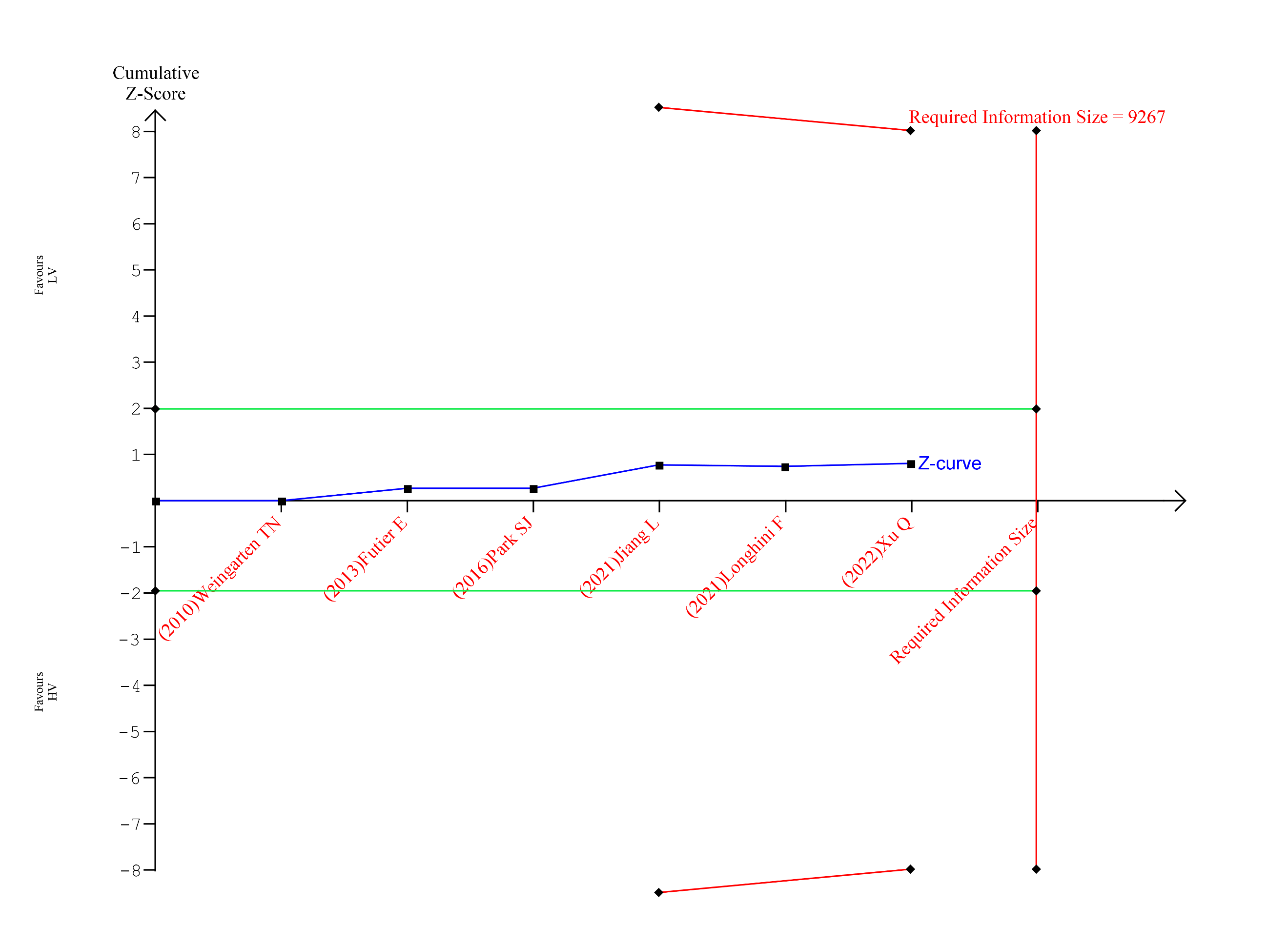

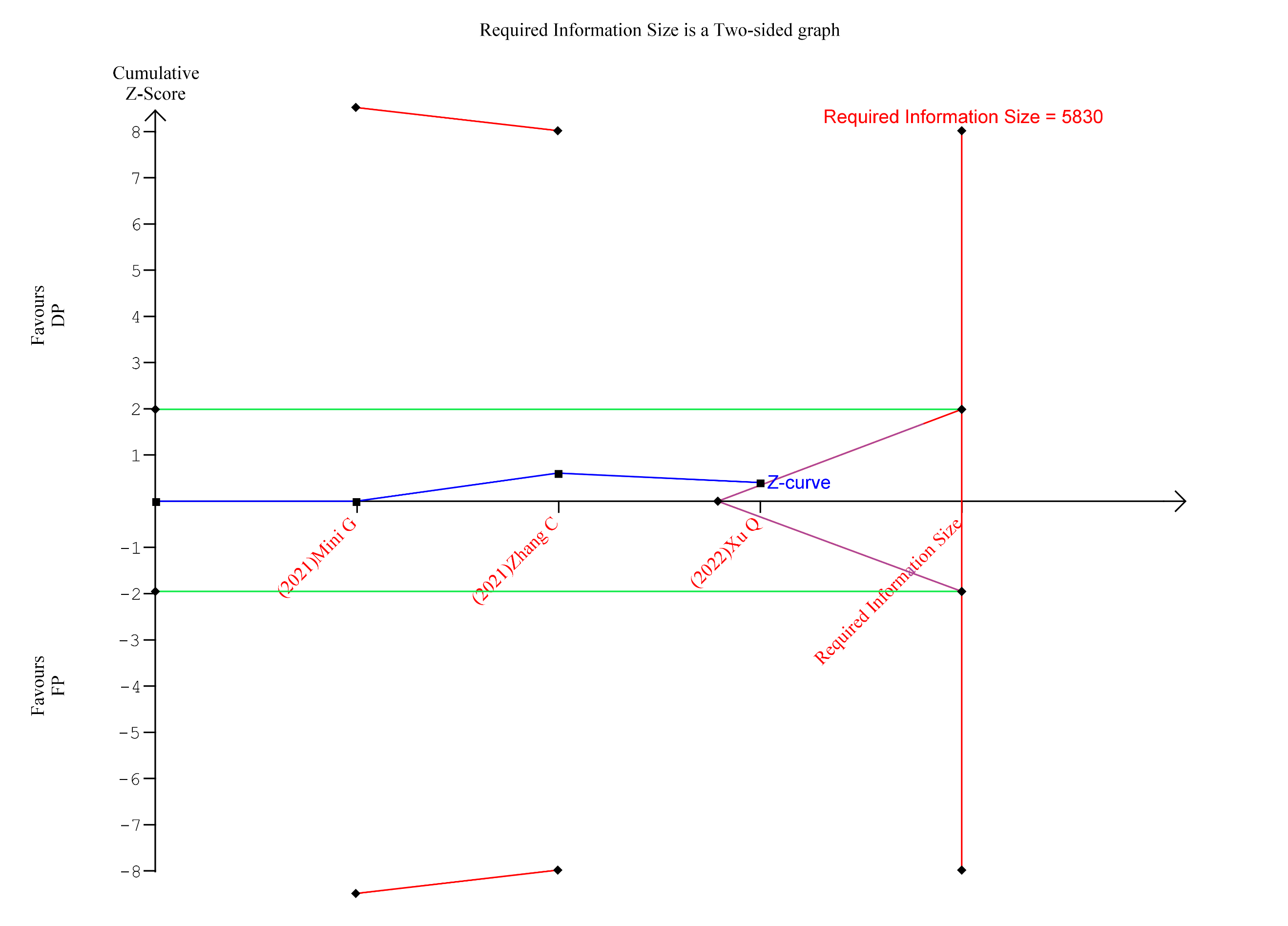


**e**

**c**

**d**

**b**

**a**

**c**

**e**

**a**

Supplemental Figure 4. TSA analysis of the different outcomes in sensitivity analysis conducted excluding studies with ‘high’ risk of bias: a) Postoperative pulmonary complications (PPCs) between low tidal volume (LV) ventilation and high tidal volume (HV) ventilation strategies; b) Cardiovascular complications (CVCs) between LV ventilation and HV ventilation strategies ; c) CVCs between driving pressure guided post-expiratory end pressure (DP) and fixed post-expiratory end pressure (FP) strategies; d) Mortality between LV ventilation and HV ventilation strategies; e) Mortality between DP and FP.


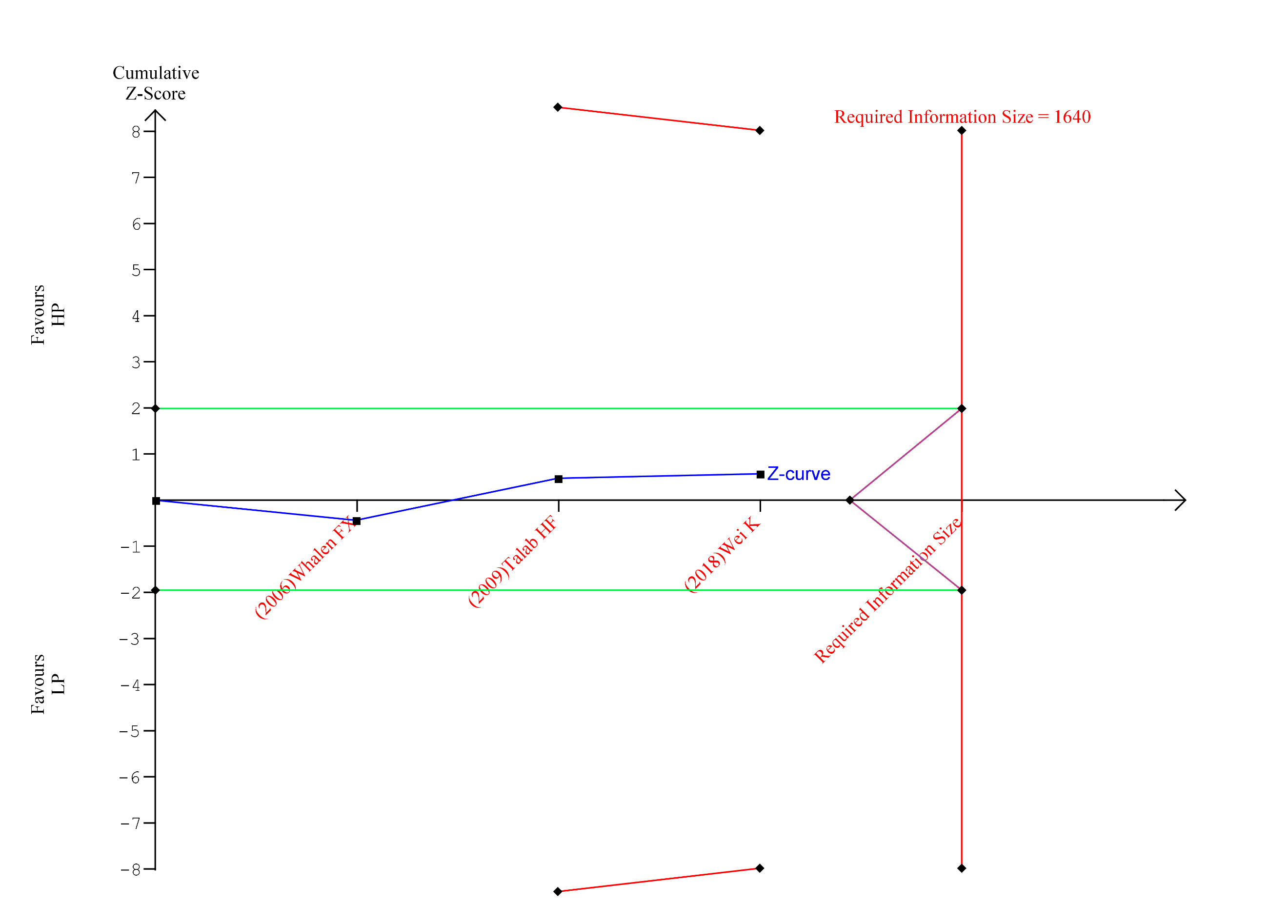

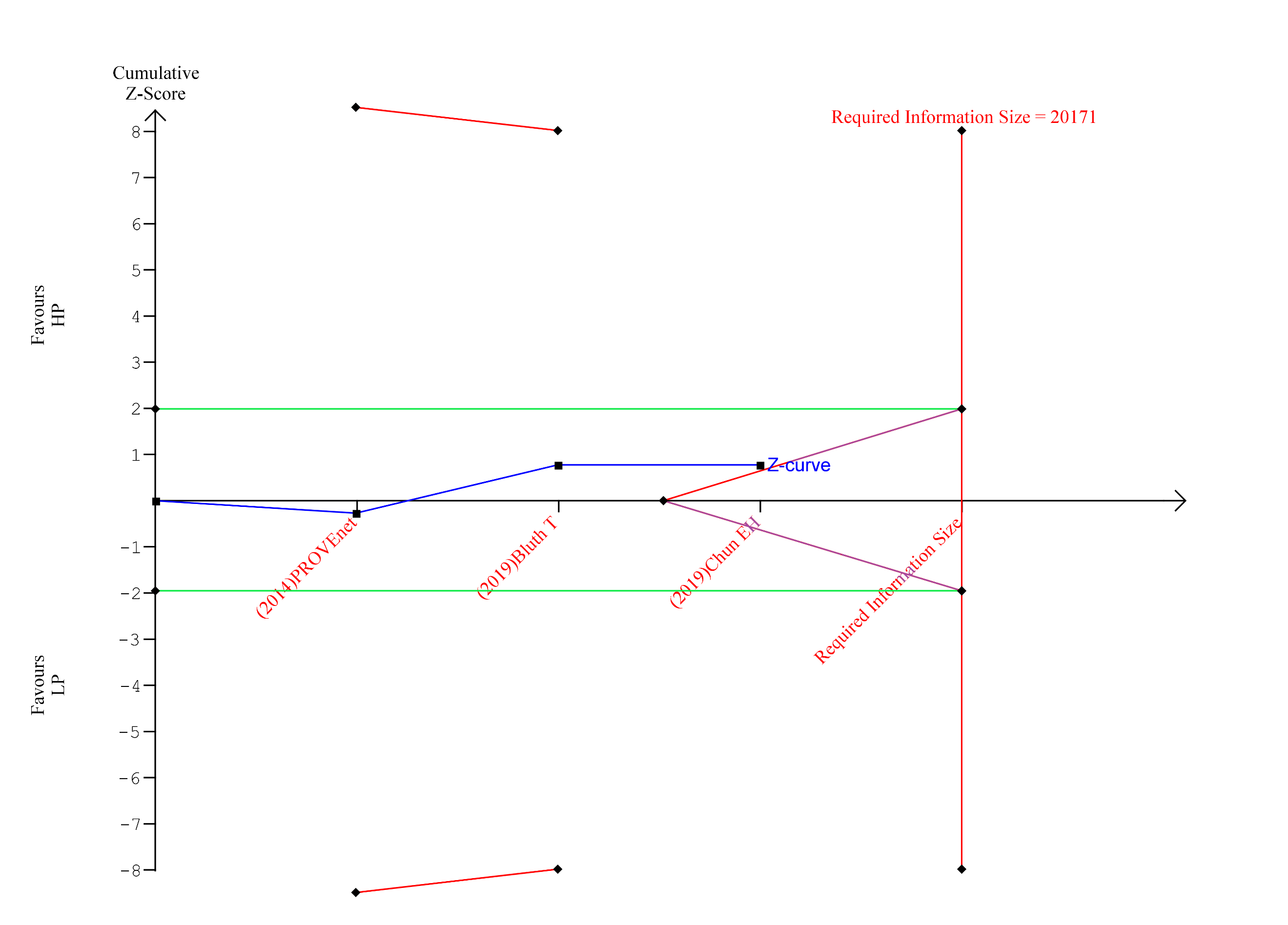

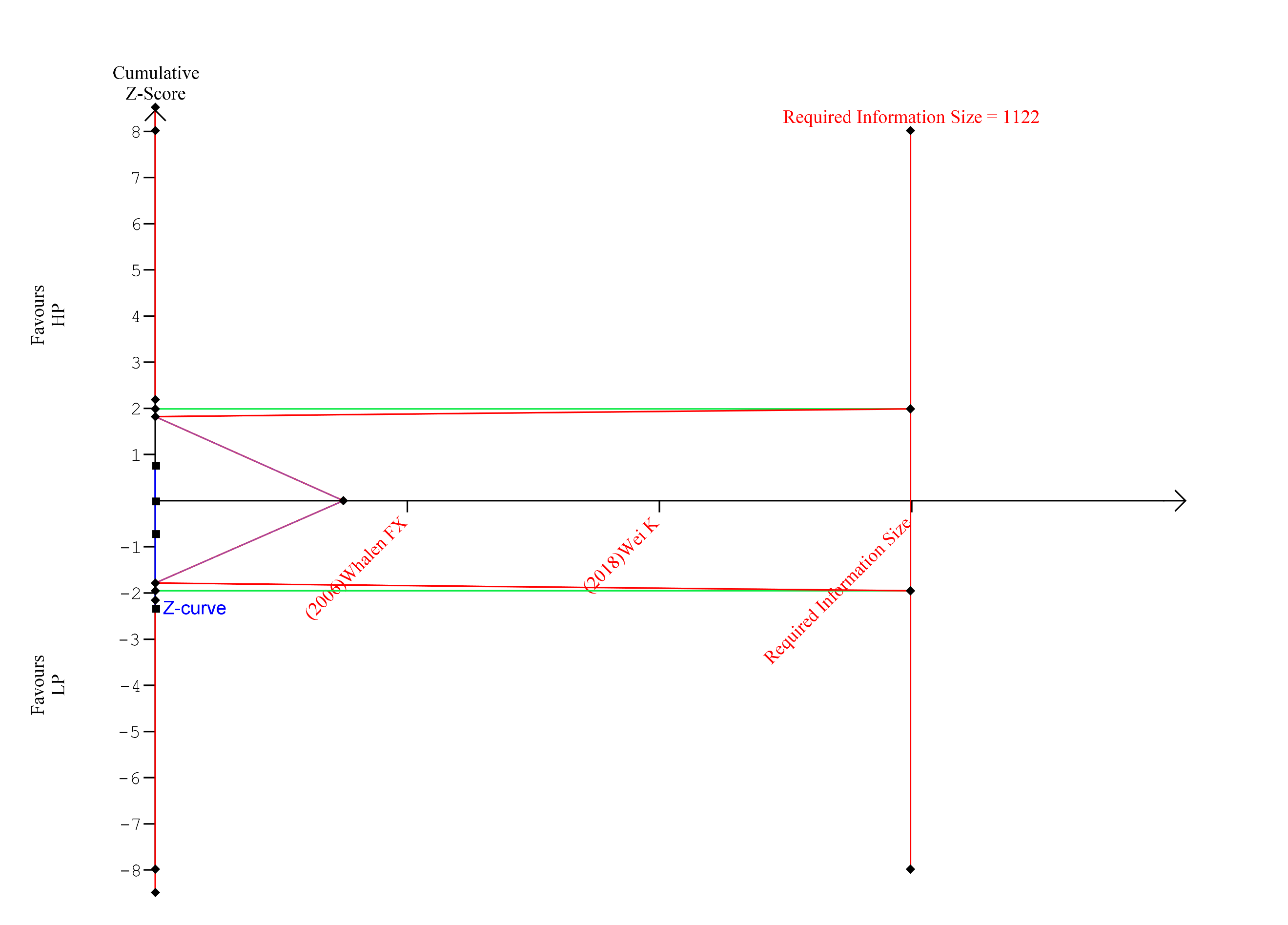

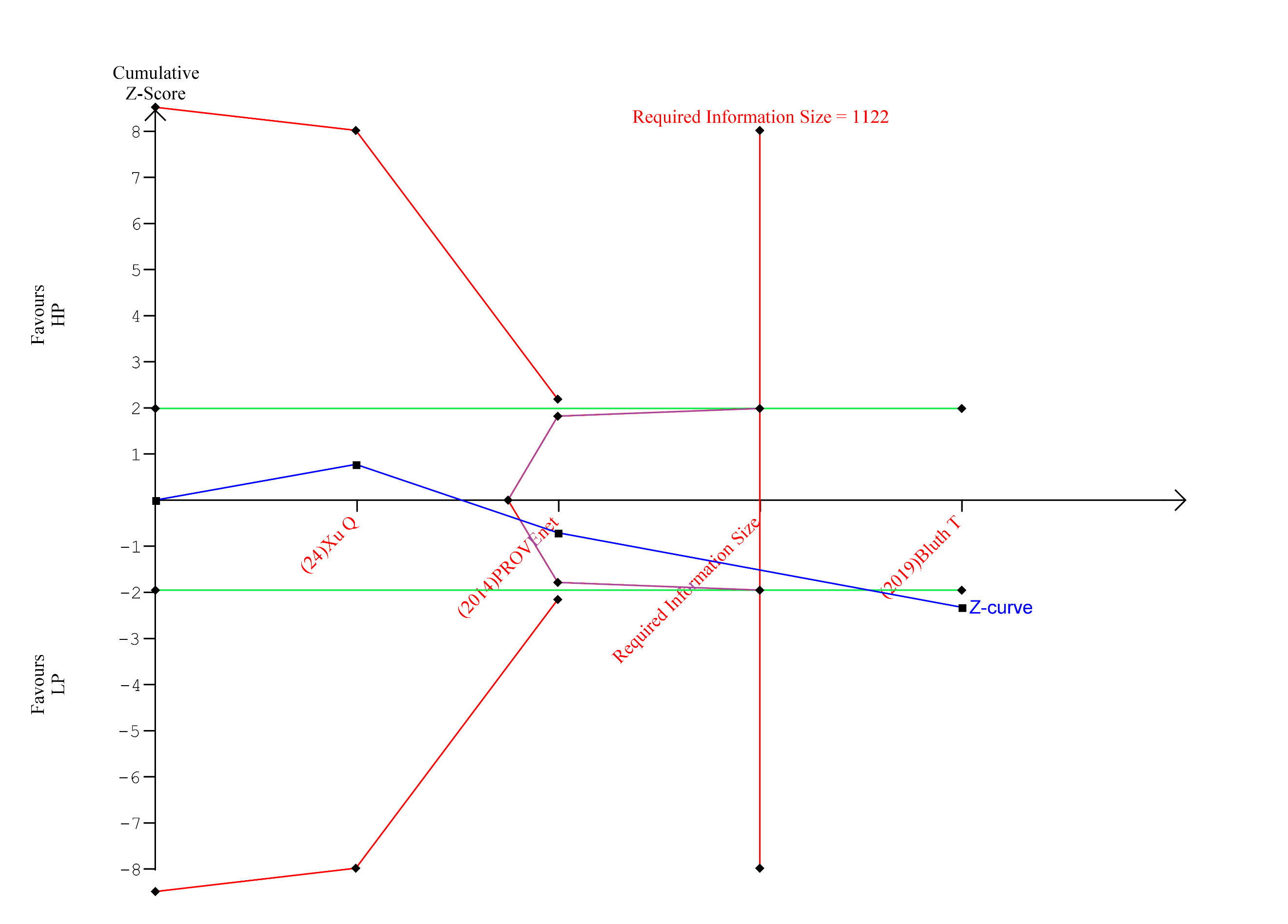


**d**

**c**

**b**

**a**


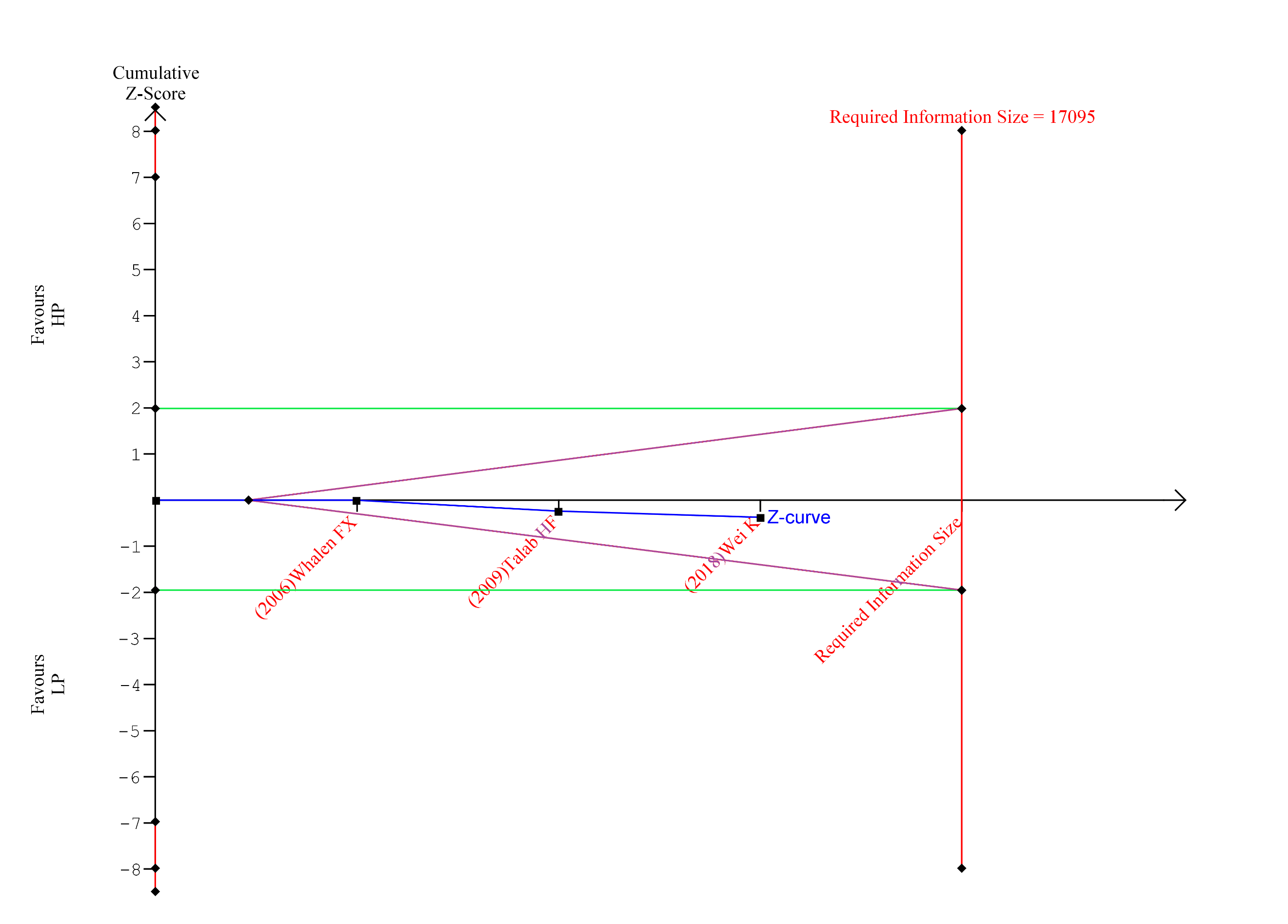

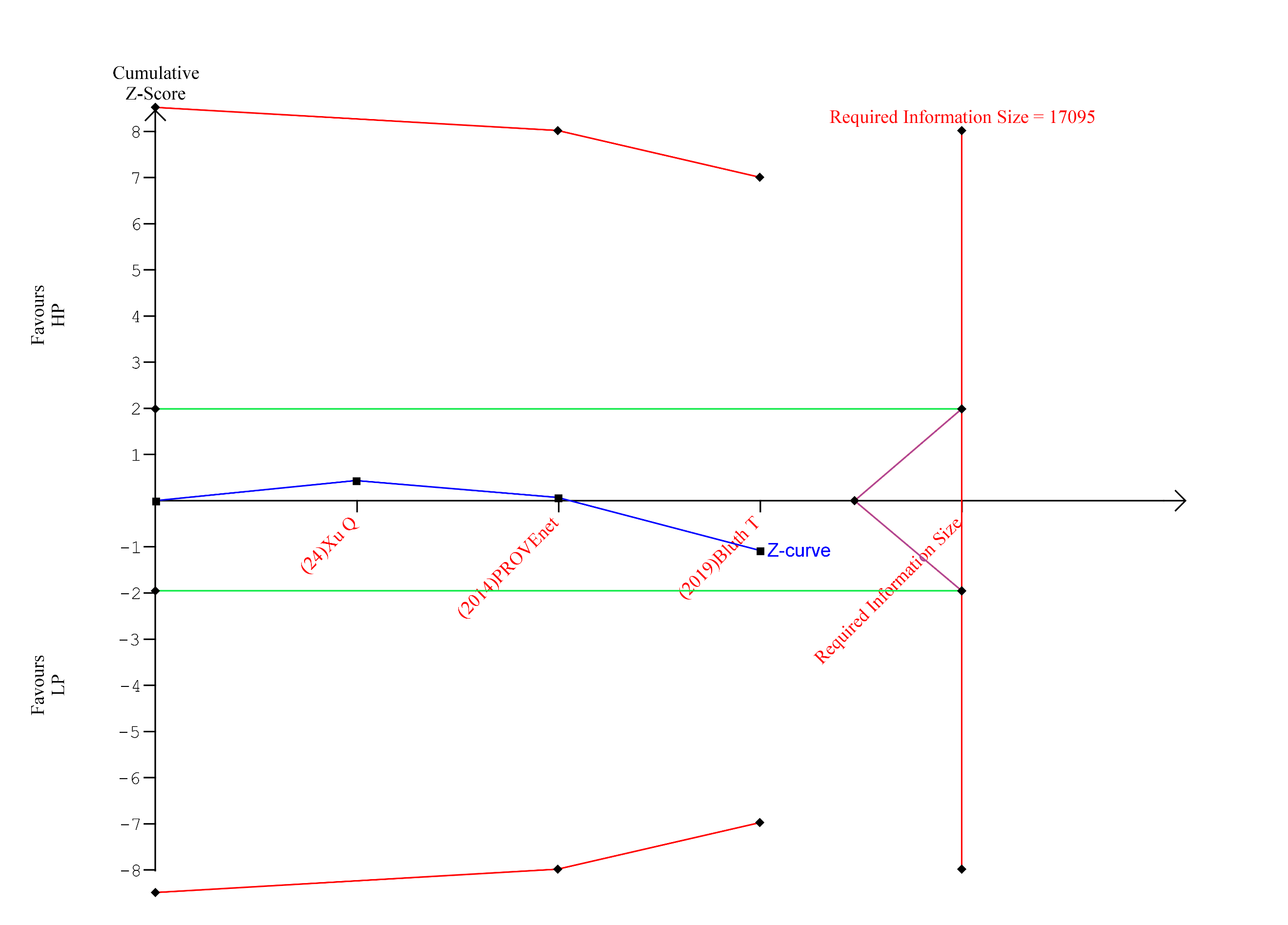


**f**

**e**

Supplementary Figure 5. TSA analysis of different outcomes between bariatric surgery (BS) and not-bariatric surgery (nBS) subgroups among the ventilation strategies: a) Postoperative pulmonary complications (PPCs) in BS subgroup among high post-expiratory end pressure (HP) and high post-expiratory end pressure (LP) strategies; b) PPCs in nBS among HP and LP strategies; c) Cardiovascular complications (CVCs) in BS subgroup among HP and LP strategies; d) CVCs in nBS subgroups among HP and LP strategies; e) Mortality in BS subgroup among HP and LP strategies; f) Mortality in nBS subgroup among HP and LP strategies
